# Supplementary material for: REducing Colonoscopies in patients without significant bowEl DiseasE: the RECEDE Study - protocol for a prospective diagnostic accuracy study
Source: BMJ Open. 2022 Mar 29;12(3):e058559. doi: 10.1136/bmjopen-2021-058559 (PMC8968545; doi:10.1136/bmjopen-2021-058559)
Supplement: Author's manuscript [file bmjopen-2021-058559.draft_revisions.pdf]

BMJ Open is committed to open peer review. As part of this commitment we make the peer review history of every article we publish publicly available.

When an article is published we post the peer reviewers' comments and the authors' responses online. We also post the versions of the paper that were used during peer review. These are the versions that the peer review comments apply to.

The versions of the paper that follow are the versions that were submitted during the peer review process. They are not the versions of record or the final published versions. They should not be cited or distributed as the published version of this manuscript.

BMJ Open is an open access journal and the full, final, typeset and author-corrected version of record of the manuscript is available on our site with no access controls, subscription charges or pay-per-view fees (http://bmjopen.bmj.com).

If you have any questions on BMJ Open's open peer review process please email info.bmjopen@bmj.com

BMJ Open

REducing Colonoscopies in patients without significant bowEl DiseasE – The RECEDE Study - Protocol for a Prospective Diagnostic Accuracy Study.

Journal:	*BMJ Open*
Manuscript ID	bmjopen-2021-058559
Article Type:	Protocol
Date Submitted by the Author:	21-Oct-2021
Complete List of Authors:	Bradley, Christopher; University Hospitals Coventry and Warwickshire NHS Trust, Research & Development<br>Hee, Siew Wan; University of Warwick,<br>Andronis, Lazaro; University of Warwick, Warwick Medical School<br>Persaud, Krishna; The University of Manchester, Department of Chemical Engineering<br>Hull, Mark A.; Univ Bradford<br>Todd, John; University Hospitals Coventry and Warwickshire NHS Trust, Research & Development<br>Taylor-Phillips, Sian; University of Warwick, Warwick Medical School<br>Smith, Steve; University Hospitals of Coventry & Warwickshire NHS Trust, Midlands & NW Bowel Cancer Screening Hub, Hospital of St. Cross,<br>Waugh, Norman; University of Warwick, Warwick Evidence<br>Constable, Rachel; University Hospitals Coventry and Warwickshire NHS Trust, Research & Development<br>Arasaradnam, Ramesh; University Hospitals Coventry and Warwickshire NHS Trust, Gastroenterology
Keywords:	Endoscopy < GASTROENTEROLOGY, Computed tomography < RADIOLOGY & IMAGING, Colorectal surgery < SURGERY

SCHOLARONE™
Manuscripts

Protocol for Publication v1.0_18.10.2021 - BMJ Open

Title: REducing Colonoscopies in patients without significant bowEl DiseasE – The RECEDE Study - Protocol for a Prospective Diagnostic Accuracy Study.

Authors:

Chris Bradley – Christopher.bradley@uhcw.nhs.uk[1]

Siew Wan Hee – S.W.Hee@warwick.ac.uk[2]

Lazaros Andronis – l.andronis@warwick.ac.uk[2]

Krishna Persaud – krishna.persaud@manchester.ac.uk[3]

Mark A Hull – m.a.hull@leeds.ac.uk[4]

John Todd

Sian Taylor-Phillips – S.taylor-phillips@warwick.ac.uk[2]

Steve Smith – steve.smith@uhcw.nhs.uk[1]

Norman Waugh norman.waugh@warwick.ac.uk[2]

Rachel Constable – Raschel.constable@uhcw.nhs.uk[1]

Ramesh Arasaradnam – Ramesh.arasaradnam@uhcw.nhs.uk[1]

Corresponding Author

Chris Bradley

University Hospitals Coventry & Warwickshire NHS Trust, Clifford Bridge Road, Coventry, CV2 2DX

Christopher.bradley@uhcw.nhs.uk

02476966197

Author Conflicts:

No competing interests declared

[1] University Hospitals Coventry & Warwickshire NHS Trust, Coventry, England
[2] University of Warwick, Coventry, England
[3] University of Manchester, Manchester, England
[4] University of Leeds, Leeds, England

For peer review only - http://bmjopen.bmj.com/site/about/guidelines.xhtml

Acknowledgements:

This study is funded by the National Institute for Health Research (NIHR) [Health Service and Delivery Research (project reference 127800)]. The views expressed are those of the authors and not necessarily those of the NIHR or the Department of Health and Social Care.

In memory of John Todd, deceased 17th April 2021

Key Words:

Colonoscopy, Computed Tomography Colonography, Volatile Organic Compounds, Faecal Immunochemical Testing, Colorectal Cancer

Word Count:

3608

For peer review only

Abstract

Introduction: Demand for colonoscopies and computed tomography colonography (CTC) is exceeding capacity in NHS Trusts. In many patients colonoscopies and CTCs show no significant bowel disease (SBD). Faecal Immunochemical Testing (FIT) is being introduced to prioritise patients for colonoscopies but is insufficient to identify non-SBD patients meaning colonoscopy and CTC demand remains high. The RECEDE study aims to test urine volatile organic compound (VOC) analysis alongside FIT to improve detection of SBD and to reduce the number of colonoscopies and CTCs.

Methods and Analysis: This is a multi-centre, prospective diagnostic accuracy study evaluating whether stool FIT plus urine VOC compared to stool FIT alone improves detection of SBD in patients referred for colonoscopy or CTC due to persistent lower gastrointestinal (GI) symptoms. To ensure SBD is not missed, the dual test requires a high sensitivity, set at 97% with 95% confidence interval width of 5%. Our assumption is that to achieve this sensitivity requires 200 participants with SBD. Further assuming 19% of all participants will have SBD and 55% of all participants will return both stool and urine samples we will recruit 1915 participants. The thresholds for FIT and VOC results diagnosing SBD have been pre-set. If either FIT or VOC exceeds the respective threshold, the participant will be classed as having suspected SBD. As an exploratory analysis we will be testing different thresholds. The reference comparator will be a complete colonoscopy or CTC. Secondary outcomes will look at optimising the FIT and VOC thresholds for SBD detection. An economic evaluation, using a denovo decision analytic model, will be carried out determine the costs, benefits and overall cost-effectiveness of FIT + VOC versus FIT followed by colonoscopy.

Ethics and Dissemination: Ethical approval was obtained by Liverpool Central Research Ethics Committee (20/NW/0346). RECEDE is registered on Clinicaltrials.gov (NCT04516785) & ISRCTN (14982373)

For peer review only - http://bmjopen.bmj.com/site/about/guidelines.xhtml
For peer review only

Article Summary

Strengths and Limitations of the Study

This is a simple trial design that does not affect the standard of care a participant would receive.

Results from the study can inform clinicians worldwide to review the patient referral pathway for patients presenting with lower GI symptoms. Patients can potentially benefit as fewer may be required to undergo a colonoscopy or CTC which is invasive and uncomfortable.

If the addition of VOC to FIT proves effective in improving SBD detection, there is the potential for a large annual savings for NHS hospitals across the UK from the use of low cost FIT and VOC testing to reduce colonoscopies, which currently cost around £525-£625, plus outpatient costs.

Since the start of the COVID-19 pandemic, referrals to gastroenterology units have dropped markedly. The number is slowly rising as COVID-19 cases fall. However this could still present a challenge to recruitment for this study. FIT could be used as an aid to prioritise patients for investigation but such use requires careful consideration of thresholds and pathways [1].

Introduction

There is currently a disparity between demand and available resources for colonoscopies and CTCs within the NHS. Before the COVID-19 pandemic, around 300,000 patients were being referred annually to NHS trusts suspected of having colorectal cancer (CRC) and this number was rising [2]. Most patients referred with suspected CRC are offered an invasive colonoscopy or CTC examination, but only 30% of these participants have significant bowel disease (SBD) [3]. SBD is defined as CRC, adenomatous polyps or inflammatory bowel disease [4]. The remaining 70% have a normal examination with 30% of those having functional conditions such as irritable bowel syndrome (IBS).

Set against this there will remain a capacity shortfall for colonoscopies and CTCs for the foreseeable future. This limits capacity of the NHS to extend CRC detection within the Bowel Cancer Screening

For peer review only - http://bmjopen.bmj.com/site/about/guidelines.xhtml

Programme (BCSP) or to investigate those who present through the emergency department. The imbalance between capacity and demand, coupled with a lack of an accurate triage test makes it difficult for the NHS to stratify patients who present with bowel symptoms to those most at risk of SBD. In the COVID-19 era, faecal immunochemical testing (FIT) is being introduced to triage patients who present with lower GI symptoms [1]. However almost all patients still receive a colonoscopy or alternative colonic imaging at some time and the optimum threshold for FIT has yet to be determined. The National Institute for Health and Care Excellence (NICE) have recommended 10μ gHb/g faeces [5], whereas the BCSP have set a cut off of 120μgHb/g faeces [6]. Even at the lower threshold some SBD cases can be missed [3, 7, 8]. Previous data have shown that FIT alone (at a threshold of 7μgHb/g faeces) has a sensitivity of 80%, 53%and 86% for CRC, adenomatous polyps and inflammatory bowel disease (IBD) respectively [3]. A lower FIT threshold may reduce the number of false negatives but it will increase the number of unnecessary colonoscopies and CTCs in patients with no abnormalities (false positives). Moreover it will always miss cases of SBD where there is no bleeding.

Thus, this study will test urine volatile organic compound (VOC) analysis alongside FIT to improve detection of SBD. VOCs are produced by metabolic responses to inflammation in the presence of illness, therefore they are disease specific [9, 10, 11]. Previous work has identified key metabolites in inflammatory GI disease [12] and, on its own, VOC analysis has a sensitivity of 80%, 92% and 86% for CRC, adenomatous polyps and IBD respectively [13, 14]. When FIT and VOC are used in combination (FIT followed by VOC in the FIT negative), sensitivity of CRC improves from 80% to 97%, which is similar to the results of colonoscopy and CTC [15, 13]. Existing studies have already demonstrated the utility of VOC analysis in detecting IBD, coeliac disease and bile acid diarrhoea whilst also being negative in functional conditions such as IBS [16, 17]. Many patients with slightly raised FIT are found to have no SBD and we will examine the value of VOC in this group to see if a negative VOC can rule out SBD.

For peer review only

For peer review only - http://bmjopen.bmj.com/site/about/guidelines.xhtml

73 Therefore, the objective of this study is to investigate whether the combination of FIT and VOC analysis compared to FIT alone improves detection of SBD in patients who present with lower GI symptoms. . This could be by both improving sensitivity in patients with FIT < 10 and but also possibly by improving specificity in patients with slightly raised FIT but negative VOC. If correct, fewer patients will be referred for colonoscopies and CTCs, freeing up capacity and reducing NHS costs, whilst reducing disutility in patients who do not benefit from an invasive examination. Within the COVID-19 era, this has added importance. If the proposed method of dual testing proves effective then not only will hospital capacity be improved but also fewer patients will be required to undergo colonoscopies or CTCs; streamlining the patient pathway and reducing the risk of viral transmission if colonoscopy generates aerosols.

83 Methods and Analysis

84 Study Design and Procedures

85 This is a multi-centre (UK only), prospective diagnostic accuracy study, sponsored by the University Hospitals Coventry & Warwickshire (UHCW) to compare dual testing of stool FIT plus urine VOC markers compared to stool FIT alone in the detection of SBD. Participants will be recruited when referred to secondary care for investigation of lower bowel symptoms, after informed consent is received. **Inclusion criteria** will be: patients referred routinely or urgently (fulfilling the national criteria for referral – NICE NG12) with lower GI symptoms and determined by the overseeing clinician to require colonoscopy or CTC; minimum age of 18; able to provide informed consent, and have the ability to return both stool and urine samples. **Exclusion criteria** are those who are pregnant. Participants will be recruited over the phone or face-to-face during a clinic visit which precedes their colonoscopy or CTC. Participants will be asked to provide a stool sample and urine sample at least 24 hours before commencing their bowel cleansing medication. Stool samples will be collected at home by the participants using FIT collection kits and posted directly to the analysis site. If the participant has already completed a FIT during their referral pathway, we will use this data if

For peer review only - http://bmjopen.bmj.com/site/about/guidelines.xhtml

the time between the FIT and their colonoscopy is less than 4 weeks, otherwise we will request that the participant to provide another FIT. Urine samples will be returned to hospitals and frozen at -80°C pending future analysis. Urine samples can either be collected during a clinic visit that precedes their colonoscopy or CTC, or collected at the home of the participant and brought back by the participant. We will aim for the time between the urine sample being produced and the urine sample being frozen at -80°C to be less than 4 hours, otherwise we will ask participants to freeze their urine samples in their home freezer. For the purposes of the economic analysis, a subset of participants (20% of total) undergoing colonoscopy or CTC will also be asked to complete questionnaires at five time points surrounding their examination. This will help determine the disutility and costs associated with undergoing colonoscopy or CTC, which will be used as inputs in the economic model (see section Economic Evaluation below). Figure 1 show's a participant flow diagram once enrolled in the RECEDE study.

Sample Size Calculation

To replace colonoscopy – the current gold standard for diagnosing bowel conditions – and/or CTC, FIT and VOC analysis requires a very high sensitivity to avoid false negatives. High specificity is also desirable, to reduce false positives. Based on our previous study, the sensitivity of FIT and VOC analysis to detect CRC was 97%, comparable to colonoscopy. Therefore, the sample size was determined to achieve a sensitivity of at least 97% with a 95% confidence interval width of 5% (Zhou-Li method [18] as described in Box 1). The required number of participants with SBD to achieve this sensitivity is 200. Figure 2 shows confidence intervals for different observed sensitivity cases of SBD (150, 200, 250). Figure 3 shows the widths of the confidence interval limits are narrower and wider when the observed sensitivity is above or below 97% [13], respectively. This sample size is also robust to negative predictive value (NPV), an outcome that is of interest as a high NPV suggests that participants without SBD would avoid having colonoscopy examination.. Assuming the prevalence of SBD in the study population is 19%, then 1053 participants are required. Based on

For peer review only

For peer review only - http://bmjopen.bmj.com/site/about/guidelines.xhtml

our previous study [13], the return rate of both stool and urine samples that are eligible for analysis is about 55% resulting in a total required sample size of 1915 participants across multiple UK sites within a 24 month period.

Stool Sample Analysis

Stool samples will be analysed for traces of haemoglobin in the faeces using a HM-JACKarc. Participants samples will be defined as having suspected SBD if the value is >10μgHb/g faeces. Any previous FIT results collected to triage patients can be sourced from patient records if the test is <4 weeks before their colonoscopy or CTC.

Urine VOC Analysis

Participants will be asked to collect duplicate urine samples in universal sterilin pots. Urine samples will be transported to the analysis site using dry ice to maintain the cold chain. All urine samples will be analysed using the ‘electronic nose’ [16] technique. Briefly, this technique enables separation of VOCs between disease groups based on chemical fingerprint pattern rather than specific chemical analysis. This allows screening of populations rapidly using chemo metric data analysis (information extracted from chemical systems) and receiver operator curves (ROC) to distinguish one population from another, with ROCs being used to measure the appropriate thresholds to be set to distinguish samples as having SBD. A sample which has a ROC with an area under the curve of 0.63 or greater will be defined as having suspected SBD.

A subset of 100 urine samples will also be analysed using a gas-chromatography mass spectroscopy system. This technique separates complex mixtures of chemicals based on their interaction with a retentive layer, resulting in chemicals eluding out of the gas-chromatography at different times. These individual chemicals are then ionised and the mass of the resultant fragments measured. The analysis will be split into two stages. Firstly, a subset of SBD positive and SBD negative samples will be screened to identify up to 8 VOCs that are prominent in SBD positive samples. Once identified, a

For peer review only - http://bmjopen.bmj.com/site/about/guidelines.xhtml

headspace gas chromatography/mass spectrometry (HSGC-MS) method will be developed to detect, separate and quantify the target VOCs selected in stage one within the remaining samples. This method will highlight whether specific VOCs present more frequently in SBD positive samples.

Adoption of Study due to COVID pandemic – remote delivery

In order to continue recruiting through the COVID-19 pandemic, an innovative study design was adopted whereby participant involvement shifted to an entirely remote approach. This allowed the study to continue recruiting through the peak waves of COVID-19 when many other studies were forced to temporarily pause [19].

Reference Test

The reference test is the final report from the colonoscopy or CTC examination and confirmatory histology report. Biopsies are required as per national guidance [5] in the investigation of participants with lower GI symptoms even in the absence of macroscopic abnormalities.

Data Management

Data from the study will be stored on an online validated GCP compliant electronic data capture (EDC) system. Individual user access will be provided only to members of the research team who require it for their role. No personal data will be uploaded to the EDC. Participant identification codes will be used to ensure pseudo-anonymization. Following the resolution of queries, the database will be locked and exported to the trial statistician for analysis. Access to the final trial dataset will only be made available to those who require it for the analysis.

Primary Outcome Analysis

The primary outcome is diagnostic accuracy (sensitivity, specificity, negative predictive value (NPV) and positive predictive value (PPV)) of the dual index test FIT and VOC analysis. The 'or' rule will be utilised for the primary analysis whereby a FIT result of >10 µgHb/g faeces OR a VOC result with

For peer review only - http://bmjopen.bmj.com/site/about/guidelines.xhtml

predicted probability >0.63 implies that the participant is suspected of having SBD and needs to be referred for colonoscopy or CTC. We will report 2x2 tables of test accuracy of the dual test by the reference standard (final report from the colonoscopy examination and confirmatory histology report of SBD which is either colon cancer, colorectal adenomas or inflammatory bowel disease), alongside summary measures of sensitivity, specificity, NPV and PPV, and their 95% confidence intervals. These test accuracy estimates will be used in the economic model.

We will analyse the combined FIT+VOC test results as both parallel (results from both tests are interpreted in combination) and serial testing (VOC analysis only performed if FIT is negative). The 'or' rule will be considered in the interpretation of both parallel and serial tests where the diagnosis is positive when either test is positive, and negative when both results are negative. In the serial test, if the FIT is positive, then the diagnosis is positive. If FIT is negative we will use the result from VOC analysis and if that is positive then the diagnosis is positive. Mathematically, both methods should give the same diagnostic results but will make a difference in the economic model.

Secondary Outcome Analyses

Secondary outcomes include: ROC of FIT plus VOC to develop an optimum thresholds for SBD detection; sensitivity, specificity, PPV and NPV estimates and their corresponding 95% CI for each individual condition of SBD (colorectal cancer, colorectal adenomas, and inflammatory bowel disease) by each individual test and combination rules; compliance rates (return of samples); the types of SBD and microscopic colitis, and the number of cases missed by CTC, FIT and VOC analyses; potential number of avoidable colonoscopies and CTCs in patients without SBD; total NHS and personal social services (PSS) costs, and total quality of adjusted life years (QALY) associated with each option. To find the optimal thresholds of FIT and VOC we will first plot the ROC curves of both tests individually to find the individual optimal thresholds (the one that gives the greatest specificity whilst achieving 97%

For peer review only - http://bmjopen.bmj.com/site/about/guidelines.xhtml

sensitivity). Secondly, we will plot combinations of the two tests in the ROC space and similarly, choose the combination of thresholds which has greatest specificity whilst achieving the required 97% sensitivity to avoid colonoscopy. The combined thresholds of FIT and VOC in the ROC space are calculated by combining every unit of FIT (from 10 to 120μgHb/g faeces) with every unit of the predicted probability of VOC (from 0.63 to 1). Both the 'or' and 'and' rules will be explored when combining the FIT and VOC results to define a case as either SBD positive or SBD negative. If both tests are in discordant then the diagnosis is negative. For each of the thresholds that we derive from the ROC curves, we will also report the NPV estimates. This will produce an overestimate of test accuracy, which we will use as a sensitivity analysis in the economic model, as a more optimistic assumption to demonstrate the range of uncertainty.

Economic Evaluation

An economic analysis will be carried out to determine the costs, benefits and overall cost-effectiveness of FIT + VOC versus FIT alone in selecting patients for colonoscopy or CTC amongst those with GI symptoms referred for investigation. The analysis will be carried out primarily from the perspective of NHS and it will employ a *de novo* analytic model built as part of this study. In line with recent literature [7], it is envisaged that the model will employ a multi-part structure, consisting of a decision tree to evaluate short-term cost and consequences accruing at the diagnosis stage followed by a state-transition model to capture the long-term (lifetime) outcomes associated with the diagnosed condition.

Calculations will consider key costs and consequences, including the costs and disutility associated with having a colonoscopy. Key model input will be drawn from the RECEDE study, including parameters related to the diagnostic characteristics of the compared options, estimates of the rate of adverse outcomes associated with colonoscopies (e.g. bowel perforation and bleeding) and CTCs, as well as primary and secondary care resource use and preference-based health-related quality of life (utility) values associated with colonoscopy. The latter information will be needed to reflect the

For peer review only - http://bmjopen.bmj.com/site/about/guidelines.xhtml

fact that undergoing a colonoscopy, including the bowel preparation and the procedure itself is an unpleasant activity which is likely to result in a temporary decrease in quality of life. Estimates of changes in quality of life will be obtained by administering a widely used and recommended generic questionnaire (EuroQol EQ-5D-5L [20]) to a sample of 370 participants (20% of the total study sample) scheduled to undergo colonoscopy or CTC at (i) baseline (i.e. when participant consent is received) (ii) immediately prior to colonoscopy once the participant has fully completed bowel preparation, (iii) 24 hours after the colonoscopy examination, (iv) 72 hours post colonoscopy and (v) 3 weeks post colonoscopy. Use of resources, including NHS care, out of pocket payments and loss of income the participants may occur as a result of their colonoscopy will also be collected through patient questionnaires administered to the same sample of participants at 3 weeks post colonoscopy or CTC.

In line with recommendations, results will be presented in terms of total costs per additional QALY associated with FIT + VOC compared to FIT alone [20]. Deterministic and probabilistic sensitivity analyses will be undertaken to explore the robustness of the obtained results to sample variability and plausible variations in key assumptions and employed analytical methods [22, 23]. The model will also form the basis for conducting value of information analysis, which will quantify the total expected cost due to the remaining uncertainty around the decision problem [24, 25]

Peer Review and Public & Participant Involvement

The RECEDE study has been peer reviewed in the NIHR HSDR process by external reviewers and the commissioning board. This protocol has also been reviewed externally by a Trial Steering Committee and internally by the Trial Management Group (TMG). The study was also reviewed by members of the GUT club (survivors of GI cancer) and the patient and public involvement in research (PPI) at the sponsor site. Both groups were supportive of the aims of RECEDE and agreed further evidence is required for the utility of FIT and VOC analysis in diagnosing SBD.

For peer review only - http://bmjopen.bmj.com/site/about/guidelines.xhtml

Ethics and Dissemination

Ethical approval was provided by Liverpool Central Research Ethics Committee (20/NW/0346). The study will be conducted in compliance with the principles of the ICH GCP guidelines and in accordance with all applicable regulatory guidance. The study will comply with the current Data Protection regulations and regular checks and monitoring will be undertaken by the study manger to ensure compliance.

Results from RECEDE will naturally be of profound interest to clinicians worldwide who investigate lower GI symptoms. Results can also be used by NICE to inform a revised pathway for managing participants with lower GI symptoms – to categorise high risk i.e. those with SBD versus low risk (without SBD). Those with very low risk of SBD could potentially be managed in primary care without need to refer to secondary care. RECEDE will lead to high level presentations at GI and Oncological meetings both nationally and internationally. It will also result in high quality open access manuscripts. A range of dissemination products will include annual reports, national publications, press releases through UHCW Communications Department, participant safety collaborations, presentation and talks as well as videos will ensure that all audiences can be updated. Dissemination of results to participants will be led by our PPI co-applicant and facilitated through GUTs UK which is the partner charity of the British Society of Gastroenterology. We will also engage with key stakeholders including PPI groups, local specialised colorectal Clinical Research Group, as well as Cancer Alliance groups

For peer review only

Author Statement

(1) Substantial contribution to design of work
(2) Drafting and revising the intellectual content
(3) Final approval of version to be published
(4) Agreement to be accountable for works published

For peer review only - http://bmjopen.bmj.com/site/about/guidelines.xhtml

Chris Bradley (1, 2, 3, 4), Siew Wan Hee (1, 2, 3, 4), Laz Andronis (1, 2, 3, 4), Krishna Persaud (1, 2, 3, 4), Mark Hull, (1, 2, 3, 4), John Todd (1, 2, 3, 4) Sian Taylor-Phillips (1, 2, 3, 4), Steve Smith (1, 2, 3, 4), Norman Waugh (1, 2, 3, 4), Rachel Constable (1, 2, 3, 4), Ramesh Arasaradnam (1, 2, 3, 4)

Figure Captions

Figure 1. Participant flow diagram for RECEDE. * indicates that the data is only being collected in 20% of participants.

Box 1. Sample size determination based on confidence interval method

Figure 2. Confidence intervals for different estimates of sensitivity for 150, 200 and 250 cases of SBD.

Figure 3. Confidence intervals for different estimates of NPV for 400,500 and 600 negative cases of SBD from 1000 samples.

For peer review only

For peer review only - http://bmjopen.bmj.com/site/about/guidelines.xhtml

References

[1] R. Arasaradnam, N. Bhala, C. Evans, J. Greenaway, R. Logan, I. Penman, E. Seward, B. Singh, S. Smith, J. Stephenson and N. Waugh, "Faecal immunochemical testing in the COVID-19 era: balancing risk and costs," *Lancet Gastroenterol Hepatol,* vol. 5, no. 8, pp. 717-719, 2020.

[2] NHS, "Waiting Times for Suspected and Diagnosed Cancer Patients," 2017. [Online]. Available: https://www.england.nhs.uk/statistics/wp-content/uploads/sites/2/2017/06/Cancer-Waiting-Times-Annual-Report-201617-1.pdf.

[3] M. Widlak, C. Thomas, C. Tomkins, S. Smith, N. O'Connel, S. Wurie, L. Burns, C. Harmston, C. Evans, C. Nwokolo, B. Singh and R. Arasaradnam, "Diagnostic accuracy of faecal biomarkers in detecting colorectal cancer and adenoma in symptomatic patients," *Aliment Pharmacol Ther,* vol. 45, no. 2, pp. 354-363, 2017.

[4] A. Adler, E. Solter, S. Masarwa, T. Miller-Roll, B. Abu-Libdeh, H. Khanmash, K. Najem, S. Dekadek, C. Stein-Zamir, N. Nubani, A. Kunbar, M. Assous, Y. Carmeli and M. Schwaber, "Epidemiological and Microbiological Characteristics of an Outbreak Caused by OXA-48-Producing Enterobacteriaceae in a Neonatal Intensive Care Unit in Jerusalem, Israe," *J Clin Microbiol,* vol. 51, no. 9, pp. 2926-2930, 2013.

[5] NICE, "Quantitive faecal immunochemical tests to guide referral for colorectal cancer in primary care," 2017. [Online]. Available: https://www.nice.or.uk/guidance/dg30.

[6] J. Murphy, S. Halloran and A. Gray, "Cost-effectiveness of the faecal immunochemical test at a range of positivity thresholds compared with the guaiac faecal occult blood test in the NHS Bowel Cancer Screening Programme in England," *BMJ Open,* vol. 7, no. 10, p. e017186, 2017.

[7] M. Westwood, I. Corro Ramos, S. Lang, M. Luyendijk, R. Zaim, L. Stirk, L. Maiwenn, N. Armstrong and J. Kleijnen, "Faecal immunochemical tests to triage participants with lower abdominal symptoms for suspected colorectal cancer referrals in primary care: a systematic review and cost-effectiveness analysis," *Health Technol Assess,* vol. 21, no. 33, pp. 1-234, 2017.

[8] J. Turvill, S. Mellen, L. Jeffery, S. Bevan, A. Keding and D. Turnock, "Diagnostic accuracy of one or two faecal haemoglobin and calprotectin measurements in participants with suspected colorectal cancer.," *Scand J Gastroenterol,* vol. 55, no. 2, pp. 69-76, 2018.

[9] A. Bannaga, K. F, K. Persaud and A. R, "Differentiating cancer types using a urine test for volatile organic compounds," *J Breath Res,* vol. 15, no. 1, p. 017102, 2020.

[10] H. Wright, B. AS, R. Iriarte, M. Mahmoud and A. R, "Utility of volatile organic compounds as a diagnostic tool in preterm infants," *Pediatr Res,* vol. 89, no. 2, pp. 263-268, 2021.

For peer review only - http://bmjopen.bmj.com/site/about/guidelines.xhtml

[11] S. Chandrapalan and R. Arasaradnam, "Urine as a biological modality for colorectal cancer detection," *Expert Rev Mol Diagn,* vol. 20, no. 5, pp. 489-496, 2020.

[12] J. Stonestreet, S. Chandrapalan, D. Woolley, U. Uthman and R. Arasaradnam, "Systematic review and meta-analysis : diagnostic accuracy of faecal immunochemical testing for haemoglobin (FIT) in detecting colorectal cancer for both symptomatic and screening population.," *Acta Gastroenterol Belg,* vol. 82, no. 2, pp. 291-299, 2019.

[13] M. Widlak, M. Neal, C. Thomas, C. Tomkins, B. Singh, C. Harmston, A. Wicaksono, C. Evans, S. Smith, R. Savage, J. Covington and R. Arasaradnam, "Risk stratification of symptomatic patients suspected of colorectal cancer using faecal and urinary markers," *Colorectal Disease,* vol. 20, no. 12, pp. 335-342, 2018.

[14] Tyagi, E. Daulton, A. Bannaga, R. Arasardnam and J. Covington, "Non-Invasive Detection and Staging of Colorectal Cancer Using a Portable Electronic Nose," *Sensor (Basel),* vol. 21, no. 16, p. 5440, 2021.

[15] S. Chandraplan, S. Bosch, J. Cubiella, J. Guardiola, P. Kimani, C. Mulder, K. Persaud, T. Meij, D. Altomare, H. Brenner, N. de Boer, L. Ricciardiello and R. Arasaradnam, "Systematic review with meta-analysis: volatile organic compound analysis to improve faecal immunochemical testing in the detection of colorectal cancer," *Aliment Pharmacol Ther,* vol. 54, no. 1, pp. 14-23, 2021.

[16] R. Arasaradnam, E. Westenbrink, M. McFarlane, R. Harbord, S. Chambers, N. O'Connell, C. Bailey, C. Nwokolo, D. Karna, R. Savage and J. Covington, "Differentiating coeliac disease from irritable bowel syndrome by urinary volatile organic compound analysis--a pilot study," *PLoS One,* vol. 9, no. 10, p. e107312, 2014.

[17] J. Covington, E. Westenbrink, N. Ouaret, R. Harbord, C. Bailey, N. O'Connell, J. Cullis, N. Williams, C. Nwokolo, K. Bardhan and R. Arasaradnam, "Application of a novel tool for diagnosing bile acid diarrhoea," *Sensors (Basel),* vol. 13, no. 9, pp. 11899-11912, 2013.

[18] N. Zhou X-HO and D. McClish, "Chapter 4: Estimation and hypothesis testing in a single sample," in *Statistical Methods in Diagnostic Medicine (Second Ed.)*, Hoboken, New Jersey, John Wiley & Sons, 2011, pp. 103-164.

[19] C. Bradley and R. Arasaradnam, "Making Research Happen in a Pandemic," National Institute of Health Research, 27 April 2021. [Online]. Available: https://www.nihr.ac.uk/blog/making-research-happen-in-a-pandemic/27549. [Accessed 18 October 2021].

[20] M. Herdman, C. Gudex, A. Lloyd, M. Janssen, P. Kind, D. Parkin, G. Bonsel and X. Badia, "Development and preliminary testing of the new five-level version of EQ-5D (EQ-5D-5L)," *Qual Life Res,* vol. 20, no. 10, pp. 1827-1736, 2011.

[21] NICE, "Guide to the methods of technology appraisal," 2013. [Online]. Available: https://www.nice.org.uk/process/pmg9/chapter/foreword.

[22] T. Takai, S. Kanaoka, K. Yoshida, Y. Hamaya, M. Ikuma, N. Miura, H. Sugimura, M. Kajimura and

For peer review only

For peer review only - http://bmjopen.bmj.com/site/about/guidelines.xhtml

A. Hishida, "Fecal cyclooxygenase 2 plus matrix metalloproteinase 7 mRNA assays as a marker for colorectal cancer screening," *Cancer Epidemiol Biomarkers Prev,* vol. 18, no. 6, pp. 1888-1893, 2009.

[23] M. Baietto and A. Wilson, "Electronic-nose applications for fruit identification, ripeness and quality grading. Sensors," *Sensors (Basel),* vol. 15, no. 2, pp. 899-931, 2015.

[24] L. Andronis, L. Billingham, S. Bryan, N. James and P. Barton, "A Practical Application of Value of Information and Prospective Payback of Research to Prioritize Evaluative Research. Med Decis Making.," *Med Decis Making,* vol. 36, no. 3, pp. 321-324, 2016.

[25] K. Claxton, L. Ginnelly, M. Sculpher, Z. Philips and S. Palmer, "A pilot study on the use of decision theory and value of information analysis as part of the NHS Health Technology Assessment programme.," *Health Technol Assess,* vol. 8, no. 31, pp. 1-103, 2004.

For peer review only

For peer review only - http://bmjopen.bmj.com/site/about/guidelines.xhtml

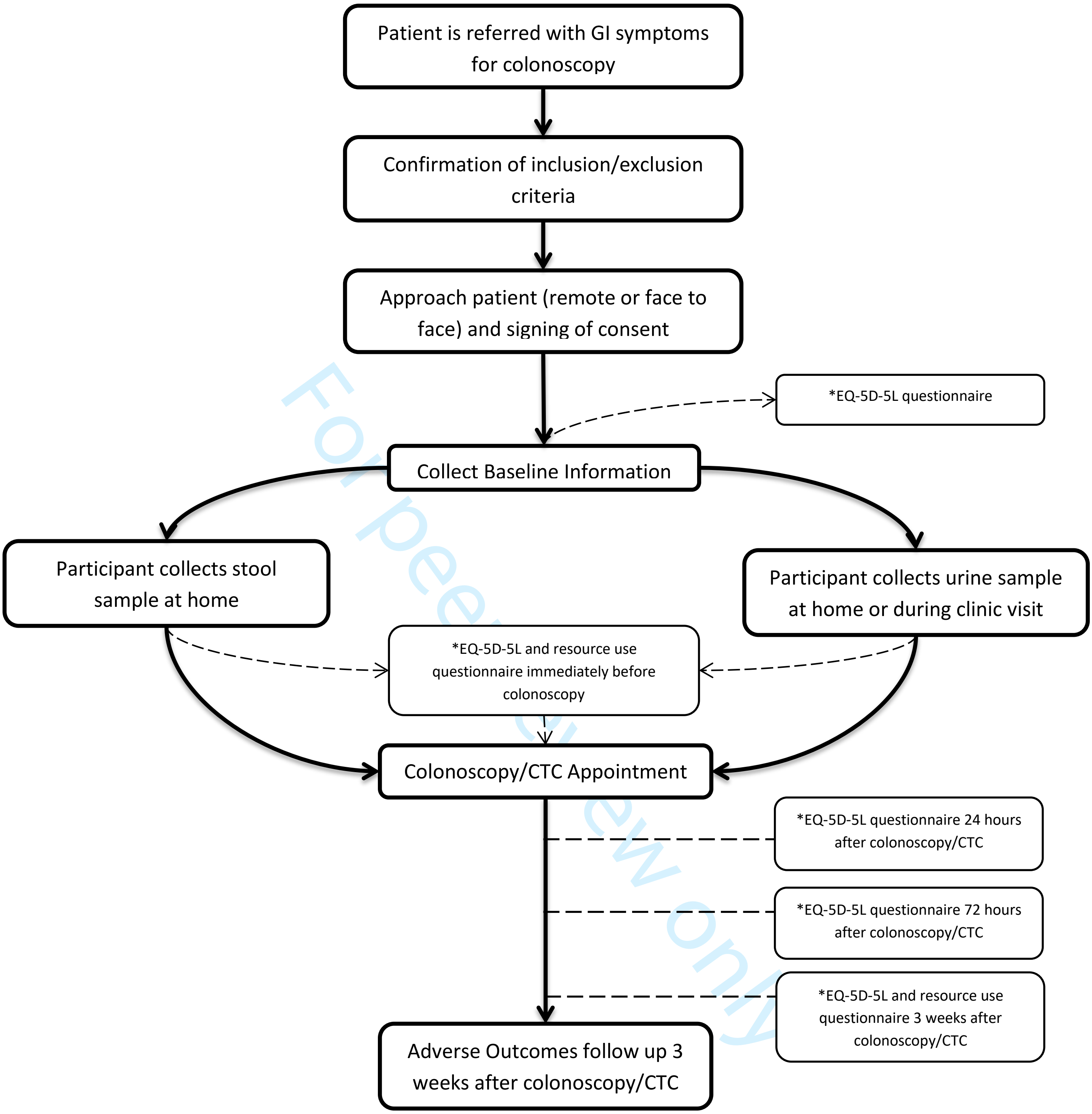

Figure 1. Participant flow diagram for RECEDE. * indicates that the data is only being collected in 20% of participants.

The Zhou-Li confidence interval is

$$\left(\frac{e^{LL}}{1+e^{LL}}, \frac{e^{UL}}{1+e^{UL}}\right)\left(\frac{e^{LL}}{1+e^{LL}}, \frac{e^{UL}}{1+e^{UL}}\right),$$

where $LL = \ln\left(\frac{\widehat{Se}}{1-\widehat{Se}}\right) - \frac{1}{\sqrt{n\widehat{Se}(1-\widehat{Se})}} g^{-1}(z_{1-\alpha/2})$, $UL = \ln\left(\frac{\widehat{Se}}{1-\widehat{Se}}\right) - \frac{1}{\sqrt{n\widehat{Se}(1-\widehat{Se})}} g^{-1}(z_{\alpha/2})$,

$g^{-1}(x) = -\sqrt{n}\left(\frac{6}{\hat{\gamma}}\right)\left\{\left[1 - \frac{\hat{\gamma}}{2}\left(\frac{x}{\sqrt{n}} - \frac{\hat{\gamma}}{6n}\right)\right]^{1/3} - 1\right\}$ and $\hat{\gamma} = \frac{1-2\widehat{Se}}{\widehat{Se}(1-\widehat{Se})}$

Box 1. Sample size determination based on confidence interval method.

For peer review only

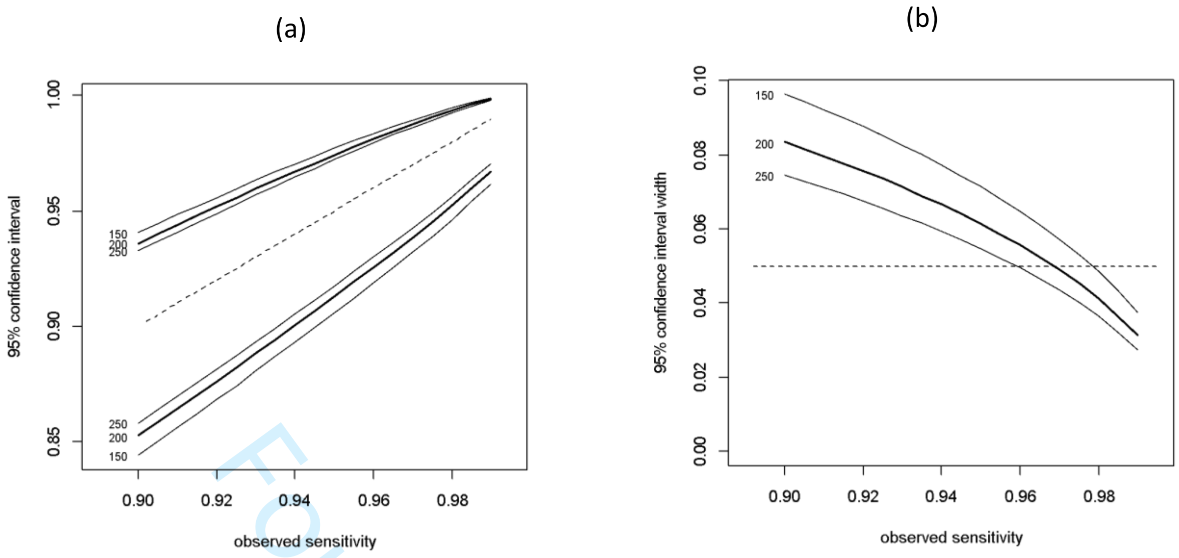

Figure 2. Confidence intervals for different estimates of sensitivity for 150, 200 and 250 cases of SBD.

For peer review only

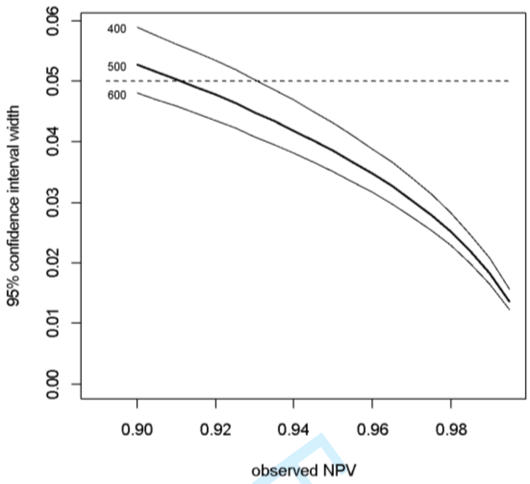

(a)

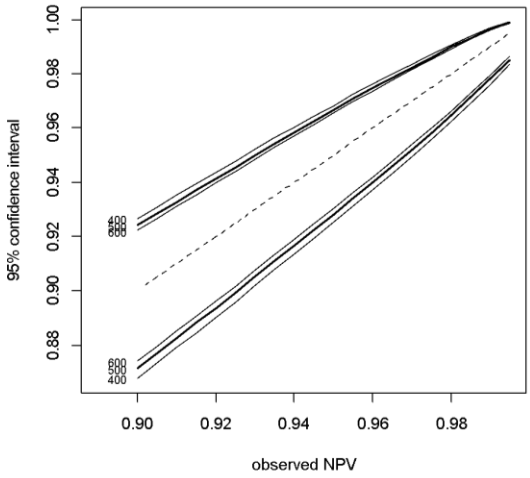

(b)

Figure 3. Confidence intervals for different estimates of NPV for 400,500 and 600 negative cases of SBD from 1000 samples.

Reporting checklist for protocol of a clinical trial.

Based on the SPIRIT guidelines.

Instructions to authors

Complete this checklist by entering the page numbers from your manuscript where readers will find each of the items listed below.

Your article may not currently address all the items on the checklist. Please modify your text to include the missing information. If you are certain that an item does not apply, please write "n/a" and provide a short explanation.

Upload your completed checklist as an extra file when you submit to a journal.

In your methods section, say that you used the SPIRITreporting guidelines, and cite them as:

Chan A-W, Tetzlaff JM, Gøtzsche PC, Altman DG, Mann H, Berlin J, Dickersin K, Hróbjartsson A, Schulz KF, Parulekar WR, Krleža-Jerić K, Laupacis A, Moher D. SPIRIT 2013 Explanation and Elaboration: Guidance for protocols of clinical trials. BMJ. 2013;346:e7586

		Reporting Item	Page Number
**Administrative information**			
Title	#1	Descriptive title identifying the study design, population, interventions, and, if applicable, trial acronym	1
Trial registration	#2a	Trial identifier and registry name. If not yet registered, name of intended registry	2
Trial registration: data set	#2b	All items from the World Health Organization Trial Registration Data Set	n/a
Protocol version	#3	Date and version identifier	1
Funding	#4	Sources and types of financial, material, and other support	1
Roles and responsibilities: contributorship	#5a	Names, affiliations, and roles of protocol contributors	12

For peer review only

Roles and responsibilities: sponsor contact information	#5b	Name and contact information for the trial sponsor	5
Roles and responsibilities: sponsor and funder	#5c	Role of study sponsor and funders, if any, in study design; collection, management, analysis, and interpretation of data; writing of the report; and the decision to submit the report for publication, including whether they will have ultimate authority over any of these activities	n/a
Roles and responsibilities: committees	#5d	Composition, roles, and responsibilities of the coordinating centre, steering committee, endpoint adjudication committee, data management team, and other individuals or groups overseeing the trial, if applicable (see Item 21a for data monitoring committee)	11
**Introduction**			
Background and rationale	#6a	Description of research question and justification for undertaking the trial, including summary of relevant studies (published and unpublished) examining benefits and harms for each intervention	3
Background and rationale: choice of comparators	#6b	Explanation for choice of comparators	4
Objectives	#7	Specific objectives or hypotheses	5
Trial design	#8	Description of trial design including type of trial (eg, parallel group, crossover, factorial, single group), allocation ratio, and framework (eg, superiority, equivalence, non-inferiority, exploratory)	5
**Methods: Participants, interventions, and outcomes**			
Study setting	#9	Description of study settings (eg, community clinic, academic hospital) and list of countries where data will be collected. Reference to where list of study sites can be obtained	5
Eligibility criteria	#10	Inclusion and exclusion criteria for participants. If applicable, eligibility criteria for study centres and individuals who will	5

For peer review only

		perform the interventions (eg, surgeons, psychotherapists)	
Interventions: description	#11a	Interventions for each group with sufficient detail to allow replication, including how and when they will be administered	5
Interventions: modifications	#11b	Criteria for discontinuing or modifying allocated interventions for a given trial participant (eg, drug dose change in response to harms, participant request, or improving / worsening disease)	n/a
Interventions: adherance	#11c	Strategies to improve adherence to intervention protocols, and any procedures for monitoring adherence (eg, drug tablet return; laboratory tests)	n/a
Interventions: concomitant care	#11d	Relevant concomitant care and interventions that are permitted or prohibited during the trial	n/a
Outcomes	#12	Primary, secondary, and other outcomes, including the specific measurement variable (eg, systolic blood pressure), analysis metric (eg, change from baseline, final value, time to event), method of aggregation (eg, median, proportion), and time point for each outcome. Explanation of the clinical relevance of chosen efficacy and harm outcomes is strongly recommended	8
Participant timeline	#13	Time schedule of enrolment, interventions (including any run-ins and washouts), assessments, and visits for participants. A schematic diagram is highly recommended (see Figure)	figure 1
Sample size	#14	Estimated number of participants needed to achieve study objectives and how it was determined, including clinical and statistical assumptions supporting any sample size calculations	6
Recruitment	#15	Strategies for achieving adequate participant enrolment to reach target sample size	8
**Methods: Assignment of interventions (for controlled trials)**			
Allocation: sequence generation	#16a	Method of generating the allocation sequence (eg, computer-generated random numbers), and list of any factors for stratification. To reduce predictability of a random sequence, details of any planned restriction (eg, blocking) should be provided in a separate document that is unavailable to those who enrol participants or assign interventions	n/a

For peer review only

Allocation concealment mechanism	#16b	Mechanism of implementing the allocation sequence (eg, central telephone; sequentially numbered, opaque, sealed envelopes), describing any steps to conceal the sequence until interventions are assigned	n/a
Allocation: implementation	#16c	Who will generate the allocation sequence, who will enrol participants, and who will assign participants to interventions	n/a
Blinding (masking)	#17a	Who will be blinded after assignment to interventions (eg, trial participants, care providers, outcome assessors, data analysts), and how	n/a
Blinding (masking): emergency unblinding	#17b	If blinded, circumstances under which unblinding is permissible, and procedure for revealing a participant's allocated intervention during the trial	n/a
**Methods: Data collection, management, and analysis**			
Data collection plan	#18a	Plans for assessment and collection of outcome, baseline, and other trial data, including any related processes to promote data quality (eg, duplicate measurements, training of assessors) and a description of study instruments (eg, questionnaires, laboratory tests) along with their reliability and validity, if known. Reference to where data collection forms can be found, if not in the protocol	12
Data collection plan: retention	#18b	Plans to promote participant retention and complete follow-up, including list of any outcome data to be collected for participants who discontinue or deviate from intervention protocols	n/a
Data management	#19	Plans for data entry, coding, security, and storage, including any related processes to promote data quality (eg, double data entry; range checks for data values). Reference to where details of data management procedures can be found, if not in the protocol	8
Statistics: outcomes	#20a	Statistical methods for analysing primary and secondary outcomes. Reference to where other details of the statistical analysis plan can be found, if not in the protocol	8
Statistics: additional analyses	#20b	Methods for any additional analyses (eg, subgroup and adjusted analyses)	9

For peer review only

Statistics: analysis population and missing data	#20c	Definition of analysis population relating to protocol non-adherence (eg, as randomised analysis), and any statistical methods to handle missing data (eg, multiple imputation)	n/a
**Methods: Monitoring**			
Data monitoring: formal committee	#21a	Composition of data monitoring committee (DMC); summary of its role and reporting structure; statement of whether it is independent from the sponsor and competing interests; and reference to where further details about its charter can be found, if not in the protocol. Alternatively, an explanation of why a DMC is not needed	n/a
Data monitoring: interim analysis	#21b	Description of any interim analyses and stopping guidelines, including who will have access to these interim results and make the final decision to terminate the trial	n/a
Harms	#22	Plans for collecting, assessing, reporting, and managing solicited and spontaneously reported adverse events and other unintended effects of trial interventions or trial conduct	n/a
Auditing	#23	Frequency and procedures for auditing trial conduct, if any, and whether the process will be independent from investigators and the sponsor	n/a
**Ethics and dissemination**			
Research ethics approval	#24	Plans for seeking research ethics committee / institutional review board (REC / IRB) approval	2
Protocol amendments	#25	Plans for communicating important protocol modifications (eg, changes to eligibility criteria, outcomes, analyses) to relevant parties (eg, investigators, REC / IRBs, trial participants, trial registries, journals, regulators)	n/a
Consent or assent	#26a	Who will obtain informed consent or assent from potential trial participants or authorised surrogates, and how (see Item 32)	5
Consent or assent: ancillary studies	#26b	Additional consent provisions for collection and use of participant data and biological specimens in ancillary studies, if applicable	n/a
Confidentiality	#27	How personal information about potential and enrolled participants will be collected, shared, and maintained in order to protect confidentiality before, during, and after the trial	5

For peer review only

Declaration of interests	#28	Financial and other competing interests for principal investigators for the overall trial and each study site	1
Data access	#29	Statement of who will have access to the final trial dataset, and disclosure of contractual agreements that limit such access for investigators	8
Ancillary and post trial care	#30	Provisions, if any, for ancillary and post-trial care, and for compensation to those who suffer harm from trial participation	n/a
Dissemination policy: trial results	#31a	Plans for investigators and sponsor to communicate trial results to participants, healthcare professionals, the public, and other relevant groups (eg, via publication, reporting in results databases, or other data sharing arrangements), including any publication restrictions	12
Dissemination policy: authorship	#31b	Authorship eligibility guidelines and any intended use of professional writers	12
Dissemination policy: reproducible research	#31c	Plans, if any, for granting public access to the full protocol, participant-level dataset, and statistical code	12
**Appendices**			
Informed consent materials	#32	Model consent form and other related documentation given to participants and authorised surrogates	n/a
Biological specimens	#33	Plans for collection, laboratory evaluation, and storage of biological specimens for genetic or molecular analysis in the current trial and for future use in ancillary studies, if applicable	7

The SPIRIT Explanation and Elaboration paper is distributed under the terms of the Creative Commons Attribution License CC-BY-NC. This checklist was completed on 19. October 2021 using https://www.goodreports.org/, a tool made by the EQUATOR Network in collaboration with Penelope.ai

For peer review only

BMJ Open

REducing Colonoscopies in patients without significant bowEl DiseasE – The RECEDE Study - Protocol for a Prospective Diagnostic Accuracy Study.

Journal:	*BMJ Open*
Manuscript ID	bmjopen-2021-058559.R1
Article Type:	Protocol
Date Submitted by the Author:	08-Dec-2021
Complete List of Authors:	Bradley, Christopher; University Hospitals Coventry and Warwickshire NHS Trust, Research & Development<br>Hee, Siew Wan; University of Warwick,<br>Andronis, Lazaro; University of Warwick, Warwick Medical School<br>Persaud, Krishna; The University of Manchester, Department of Chemical Engineering<br>Hull, Mark A.; Univ Bradford<br>Todd, John; University Hospitals Coventry and Warwickshire NHS Trust, Research & Development<br>Taylor-Phillips, Sian; University of Warwick, Warwick Medical School<br>Smith, Steve; University Hospitals of Coventry & Warwickshire NHS Trust, Midlands & NW Bowel Cancer Screening Hub, Hospital of St. Cross,<br>Waugh, Norman; University of Warwick, Warwick Evidence<br>Constable, Rachel; University Hospitals Coventry and Warwickshire NHS Trust, Research & Development<br>Arasaradnam, Ramesh; University Hospitals Coventry and Warwickshire NHS Trust, Gastroenterology
**Primary Subject Heading**:	Gastroenterology and hepatology
Secondary Subject Heading:	Research methods, Public health
Keywords:	Endoscopy < GASTROENTEROLOGY, Computed tomography < RADIOLOGY & IMAGING, Colorectal surgery < SURGERY

SCHOLARONE™
Manuscripts

Protocol for Publication v2.0_26.11.2021 - BMJ Open

Title: REducing Colonoscopies in patients without significant bowEl DiseasE – The RECEDE Study - Protocol for a Prospective Diagnostic Accuracy Study.

Authors:

Chris Bradley – Christopher.bradley@uhcw.nhs.uk[1]

Siew Wan Hee – S.W.Hee@warwick.ac.uk[2]

Lazaros Andronis – l.andronis@warwick.ac.uk[2]

Krishna Persaud – krishna.persaud@manchester.ac.uk[3]

Mark A Hull – m.a.hull@leeds.ac.uk[4]

John Todd[1]

Sian Taylor-Phillips – S.taylor-phillips@warwick.ac.uk[2]

Steve Smith – steve.smith@uhcw.nhs.uk[1]

Norman Waugh norman.waugh@warwick.ac.uk[2]

Rachel Constable – Rachel.constable@uhcw.nhs.uk[1]

Ramesh Arasaradnam – Ramesh.arasaradnam@uhcw.nhs.uk[1,2,5,6]

Corresponding Author

Chris Bradley

University Hospitals Coventry & Warwickshire NHS Trust, Clifford Bridge Road, Coventry, CV2 2DX

Christopher.bradley@uhcw.nhs.uk

02476966197

Author Conflicts:

[1] University Hospitals Coventry & Warwickshire NHS Trust, Coventry, England
[2] University of Warwick, Coventry, England
[3] University of Manchester, Manchester, England
[4] University of Leeds, Leeds, England
[5] Leicester Cancer Research Centre, University of Leicester, ENgland
[6] School of Health Sciences, University of Coventry, England

For peer review only - http://bmjopen.bmj.com/site/about/guidelines.xhtml

No competing interests declared

Acknowledgements:

This study is funded by the National Institute for Health Research (NIHR) [Health Service and Delivery Research (project reference 127800)]. The views expressed are those of the authors and not necessarily those of the NIHR or the Department of Health and Social Care.

In memory of John Todd, deceased 17th April 2021

Key Words:

Colonoscopy, Computed Tomography Colonography, Volatile Organic Compounds, Faecal Immunochemical Testing, Colorectal Cancer

Word Count:

3566

For peer review only

Abstract

Introduction: Demand for colonoscopies and computed tomography colonography (CTC) is exceeding capacity in NHS Trusts. In many patients colonoscopies and CTCs show no significant bowel disease (SBD). Faecal Immunochemical Testing (FIT) is being introduced to prioritise patients for colonoscopies but is insufficient to identify non-SBD patients meaning colonoscopy and CTC demand remains high. The RECEDE study aims to test urine volatile organic compound (VOC) analysis alongside FIT to improve detection of SBD and to reduce the number of colonoscopies and CTCs.

Methods and Analysis: This is a multi-centre, prospective diagnostic accuracy study evaluating whether stool FIT plus urine VOC compared to stool FIT alone improves detection of SBD in patients referred for colonoscopy or CTC due to persistent lower gastrointestinal (GI) symptoms. To ensure SBD is not missed, the dual test requires a high sensitivity, set at 97% with 95% confidence interval width of 5%. Our assumption is that to achieve this sensitivity requires 200 participants with SBD. Further assuming 19% of all participants will have SBD and 55% of all participants will return both stool and urine samples we will recruit 1915 participants. The thresholds for FIT and VOC results diagnosing SBD have been pre-set. If either FIT or VOC exceeds the respective threshold, the participant will be classed as having suspected SBD. As an exploratory analysis we will be testing different thresholds. The reference comparator will be a complete colonoscopy or CTC. Secondary outcomes will look at optimising the FIT and VOC thresholds for SBD detection. An economic evaluation, using a denovo decision analytic model, will be carried out determine the costs, benefits and overall cost-effectiveness of FIT + VOC versus FIT followed by colonoscopy.

Ethics and Dissemination: Ethical approval was obtained by Liverpool Central Research Ethics Committee (20/NW/0346). RECEDE is registered on Clinicaltrials.gov (NCT04516785) & ISRCTN (14982373)

For peer review only

Article Summary

Strengths and Limitations of the Study

- Simple observational trial design that does not affect the standard care of participants
- The study can be conducted entirely remotely.
- Reliance on patients collecting their samples at home and posting/bringing their samples back to the hospital may result in a higher proportion of missed samples as there is a greater chance of patients forgetting.
- Recruitment rate is dependent on COVID 19 as referral rates drop when COVID 19 cases are high [1]

Introduction

There is currently a disparity between demand and available resources for colonoscopies and CTCs within the NHS. Before the COVID-19 pandemic, around 300,000 patients were being referred annually to NHS trusts suspected of having colorectal cancer (CRC) and this number was rising [2]. Most patients referred with suspected CRC are offered an invasive colonoscopy or CTC examination, but only 30% of these participants have significant bowel disease (SBD) [3]. SBD is defined as CRC, adenomatous polyps or inflammatory bowel disease [4]. The remaining 70% have a normal examination with 30% of those having functional conditions such as irritable bowel syndrome (IBS).

Set against this there will remain a capacity shortfall for colonoscopies and CTCs for the foreseeable future. This limits capacity of the NHS to extend CRC detection within the Bowel Cancer Screening Programme (BCSP) or to investigate those who present through the emergency department. The imbalance between capacity and demand, coupled with a lack of an accurate triage test makes it difficult for the NHS to stratify patients who present with bowel symptoms to those most at risk of

For peer review only - http://bmjopen.bmj.com/site/about/guidelines.xhtml

SBD. In the COVID-19 era, faecal immunochemical testing (FIT) is being introduced to triage patients who present with lower GI symptoms [1]. However almost all patients still receive a colonoscopy or alternative colonic imaging at some time and the optimum threshold for FIT has yet to be determined. The National Institute for Health and Care Excellence (NICE) have recommended 10μgHb/g faeces [5], whereas the BCSP have set a cut off of 120μgHb/g faeces [6]. Even at the lower threshold some SBD cases can be missed [3, 7, 8]. Previous data have shown that FIT alone (at a threshold of 7μgHb/g faeces) has a sensitivity of 80%, 53%and 86% for CRC, adenomatous polyps and inflammatory bowel disease (IBD) respectively [3]. A lower FIT threshold may reduce the number of false negatives but it will increase the number of unnecessary colonoscopies and CTCs in patients with no abnormalities (false positives). Moreover it will always miss cases of SBD where there is no bleeding, and a high proportion of malignant tumours never bleed.

Thus, this study will test urine volatile organic compound (VOC) analysis alongside FIT to improve detection of SBD. VOCs are produced by metabolic responses to inflammation in the presence of illness, therefore they are disease specific [9, 10, 11]. Previous work has identified key metabolites in inflammatory GI disease [12] and, on its own, VOC analysis has a sensitivity of 80%, 92% and 86% for CRC, adenomatous polyps and IBD respectively [13, 14]. When FIT and VOC are used in combination (FIT followed by VOC in the FIT negative), sensitivity of CRC improves from 80% to 97%, which is similar to the results of colonoscopy and CTC [15, 13]. Existing studies have already demonstrated the utility of VOC analysis in detecting IBD, coeliac disease and bile acid diarrhoea whilst also being negative in functional conditions such as IBS [16, 17]. Many patients with slightly raised FIT are found to have no SBD and we will examine the value of VOC in this group to see if a negative VOC can rule out SBD.

Therefore, the objective of this study is to investigate whether the combination of FIT and VOC analysis compared to FIT alone improves detection of SBD in patients who present with lower GI symptoms. . This could be by both improving sensitivity in patients with FIT < 10 and but also

For peer review only

For peer review only - http://bmjopen.bmj.com/site/about/guidelines.xhtml

possibly by improving specificity in patients with slightly raised FIT but negative VOC. If correct, fewer patients will be referred for colonoscopies and CTCs, freeing up capacity and reducing NHS costs, whilst reducing disutility in patients who do not benefit from an invasive examination. Within the COVID-19 era, this has added importance. If the proposed method of dual testing proves effective then not only will hospital capacity be improved but also fewer patients will be required to undergo colonoscopies or CTCs; streamlining the patient pathway and reducing the risk of viral transmission if colonoscopy generates aerosols.

For peer review only

Methods and Analysis

Study Design and Procedures

This is a multi-centre (UK only), prospective diagnostic accuracy study, sponsored by the University Hospitals Coventry & Warwickshire (UHCW) to compare dual testing of stool FIT plus urine VOC markers compared to stool FIT alone in the detection of SBD. Participants will be recruited when referred to secondary care for investigation of lower bowel symptoms, after informed consent is received. **Inclusion criteria** will be: patients referred non-urgently or urgently (fulfilling the national criteria for referral – NICE NG12) [18] with lower GI symptoms and determined by the overseeing clinician to require colonoscopy or CTC; minimum age of 18; able to provide informed consent, and have the ability to return both stool and urine samples. **Exclusion criteria** are those who are pregnant. Participants will be recruited over the phone or face-to-face during a clinic visit which precedes their colonoscopy or CTC. Participants will be asked to provide a stool sample and urine sample at least 24 hours before commencing their bowel cleansing medication. Stool samples will be collected at home by the participants using FIT collection kits and posted directly to the analysis site. If the participant has already completed a FIT during their referral pathway, we will use this data if the time between the FIT and their colonoscopy is less than 4 weeks, otherwise we will request that the participant to provide another FIT. Urine samples will be returned to hospitals and frozen at -80°C pending future analysis. Urine samples can either be collected during a clinic visit that precedes

For peer review only - http://bmjopen.bmj.com/site/about/guidelines.xhtml

98 their colonoscopy or CTC, or collected at the home of the participant and brought back by the participant. We will aim for the time between the urine sample being produced and the urine sample being frozen at -80°C to be less than 4 hours, otherwise we will ask participants to freeze their urine samples in their home freezer. For the purposes of the economic analysis, a subset of participants (20% of total) undergoing colonoscopy or CTC will also be asked to complete questionnaires at five time points surrounding their examination. This will help determine the disutility and costs associated with undergoing colonoscopy or CTC, which will be used as inputs in the economic model (see section Economic Evaluation below). Figure 1 show's a participant flow diagram once enrolled in the RECEDE study.

Sample Size Calculation

To replace colonoscopy – the current gold standard for diagnosing bowel conditions – and/or CTC, FIT and VOC analysis requires a very high sensitivity to avoid false negatives. High specificity is also desirable, to reduce false positives. Based on our previous study, the sensitivity of FIT and VOC analysis to detect CRC was 97%, comparable to colonoscopy. Therefore, the sample size was determined to achieve a sensitivity of at least 97% with a 95% confidence interval width of 5% (Zhou-Li method [19] as described in Box 1). The required number of participants with SBD to achieve this sensitivity is 200. Figure 2 shows confidence intervals for different observed sensitivity cases of SBD (150, 200, 250). Figure 3 shows the widths of the confidence interval limits are narrower and wider when the observed sensitivity is above or below 97% [13], respectively. This sample size is also robust to negative predictive value (NPV), an outcome that is of interest as a high NPV suggests that participants without SBD would avoid having colonoscopy examination.. Assuming the prevalence of SBD in the study population is 19%, then 1053 participants are required. Based on our previous study [13], the return rate of both stool and urine samples that are eligible for analysis is about 55% resulting in a total required sample size of 1915 participants across multiple UK sites within a 24 month period.

For peer review only - http://bmjopen.bmj.com/site/about/guidelines.xhtml

The Zhou Li confidence interval is $\left(\frac{e^{LL}}{1+e^{LL}}, \frac{e^{UL}}{1+e^{UL}}\right)\left(\frac{e^{LL}}{1+e^{LL}}, \frac{e^{UL}}{1+e^{UL}}\right)$ where $LL = \ln\left(\frac{\widehat{Se}}{1-\widehat{Se}}\right) - \frac{1}{\sqrt{n\widehat{Se}(1-\widehat{Se})}} g^{-1}(z_{1-\alpha/2})$, $UL = \ln\left(\frac{\widehat{Se}}{1-\widehat{Se}}\right) - \frac{1}{\sqrt{n\widehat{Se}(1-\widehat{Se})}} g^{-1}(z_{\alpha/2})$, $g^{-1}(x) = -\sqrt{n}\left(\frac{6}{\hat{\gamma}}\right)\left\{\left[1 - \frac{\hat{\gamma}}{2}\left(\frac{x}{\sqrt{n}} - \frac{\hat{\gamma}}{6n}\right)\right]^{1/3} - 1\right\}$ and $\hat{\gamma} = \frac{1-2\widehat{Se}}{\widehat{Se}(1-\widehat{Se})}$.

Box 1: Sample size determination based on confidence interval method

Stool Sample Analysis

Stool samples will be analysed for traces of haemoglobin in the faeces using a HM-JACKarc. Participants samples will be defined as having suspected SBD if the value is >10μgHb/g faeces. Any previous FIT results collected to triage patients can be sourced from patient records if the test is <4 weeks before their colonoscopy or CTC.

Urine VOC Analysis

Participants will be asked to collect duplicate urine samples in universal sterilin pots. Urine samples will be transported to the analysis site using dry ice to maintain the cold chain. All urine samples will be analysed using the 'electronic nose' [16] technique. Briefly, this technique enables separation of VOCs between disease groups based on chemical fingerprint pattern rather than specific chemical analysis. This allows screening of populations rapidly using chemo metric data analysis (information extracted from chemical systems) and receiver operator curves (ROC) to distinguish one population from another, with ROCs being used to measure the appropriate thresholds to be set to distinguish samples as having SBD. A sample which has a ROC with an area under the curve of 0.63 or greater will be defined as having suspected SBD.

A subset of 100 urine samples will also be analysed using a gas-chromatography mass spectroscopy system. This technique separates complex mixtures of chemicals based on their interaction with a retentive layer, resulting in chemicals eluding out of the gas-chromatography at different times.

For peer review only - http://bmjopen.bmj.com/site/about/guidelines.xhtml

These individual chemicals are then ionised and the mass of the resultant fragments measured. The analysis will be split into two stages. Firstly, a subset of SBD positive and SBD negative samples will be screened to identify up to 8 VOCs that are prominent in SBD positive samples. Once identified, a headspace gas chromatography/mass spectrometry (HSGC-MS) method will be developed to detect, separate and quantify the target VOCs selected in stage one within the remaining samples. This method will highlight whether specific VOCs present more frequently in SBD positive samples.

Adoption of Study due to COVID pandemic – remote delivery

In order to continue recruiting through the COVID-19 pandemic, an innovative study design was adopted whereby participant involvement shifted to an entirely remote approach. This allowed the study to continue recruiting through the peak waves of COVID-19 when many other studies were forced to temporarily pause [20].

Reference Test

The reference test is the final report from the colonoscopy or CTC examination and confirmatory histology report. Biopsies are required as per national guidance [5] in the investigation of participants with lower GI symptoms even in the absence of macroscopic abnormalities. Those who perform the reference test will have no knowledge of the results of the index tests, and vice versa.

Data Management

Data from the study will be stored on an online validated GCP compliant electronic data capture (EDC) system. Individual user access will be provided only to members of the research team who require it for their role. No personal data will be uploaded to the EDC. Participant identification codes will be used to ensure pseudo-anonymization. Following the resolution of queries, the database will be locked and exported to the trial statistician for analysis. Access to the final trial dataset will only be made available to those who require it for the analysis.

For peer review only - http://bmjopen.bmj.com/site/about/guidelines.xhtml

Primary Outcome Analysis

The primary outcome is diagnostic accuracy (sensitivity, specificity, negative predictive value (NPV) and positive predictive value (PPV)) of the dual index test FIT and VOC analysis. The 'or' rule will be utilised for the primary analysis whereby a FIT result of >10 μgHb/g faeces OR a VOC result with predicted probability >0.63 implies that the participant is suspected of having SBD and needs to be referred for colonoscopy or CTC. We will report 2x2 tables of test accuracy of the dual test by the reference standard (final report from the colonoscopy examination and confirmatory histology report of SBD which is either colon cancer, colorectal adenomas or inflammatory bowel disease), alongside summary measures of sensitivity, specificity, NPV and PPV, and their 95% confidence intervals. These test accuracy estimates will be used in the economic model.

We will analyse the combined FIT+VOC test results as both parallel (results from both tests are interpreted in combination) and serial testing (VOC analysis only performed if FIT is negative). The 'or' rule will be considered in the interpretation of both parallel and serial tests where the diagnosis is positive when either test is positive, and negative when both results are negative. In the serial test, if the FIT is positive, then the diagnosis is positive. If FIT is negative we will use the result from VOC analysis and if that is positive then the diagnosis is positive. Mathematically, both methods should give the same diagnostic results but will make a difference in the economic model.

Secondary Outcome Analyses

Secondary outcomes include: ROC of FIT plus VOC to develop an optimum thresholds for SBD detection; sensitivity, specificity, PPV and NPV estimates and their corresponding 95% CI for each individual condition of SBD (colorectal cancer, colorectal adenomas, and inflammatory bowel disease) by each individual test and combination rules; compliance rates (return of samples); the types of SBD and microscopic colitis, and the number of cases missed by CTC, FIT and VOC analyses; potential number of avoidable colonoscopies and

For peer review only - http://bmjopen.bmj.com/site/about/guidelines.xhtml

CTCs in patients without SBD; total NHS and personal social services (PSS) costs, and total quality of adjusted life years (QALY) associated with each option. To find the optimal thresholds of FIT and VOC we will first plot the ROC curves of both tests individually to find the individual optimal thresholds (the one that gives the greatest specificity whilst achieving 97% sensitivity). Secondly, we will plot combinations of the two tests in the ROC space and similarly, choose the combination of thresholds which has greatest specificity whilst achieving the required 97% sensitivity to avoid colonoscopy. The combined thresholds of FIT and VOC in the ROC space are calculated by combining every unit of FIT (from 10 to 120µgHb/g faeces) with every unit of the predicted probability of VOC (from 0.63 to 1). Both the 'or' and 'and' rules will be explored when combining the FIT and VOC results to define a case as either SBD positive or SBD negative. If both tests are in discordant then the diagnosis is negative. For each of the thresholds that we derive from the ROC curves, we will also report the NPV estimates. This will produce an overestimate of test accuracy, which we will use as a sensitivity analysis in the economic model, as a more optimistic assumption to demonstrate the range of uncertainty.

Economic Evaluation

An economic analysis will be carried out to determine the costs, benefits and overall cost-effectiveness of FIT + VOC versus FIT alone in selecting patients for colonoscopy or CTC amongst those with GI symptoms referred for investigation. The analysis will be carried out primarily from the perspective of NHS and it will employ a *de novo* analytic model built as part of this study. In line with recent literature [7], it is envisaged that the model will employ a multi-part structure, consisting of a decision tree to evaluate short-term cost and consequences accruing at the diagnosis stage followed by a state-transition model to capture the long-term (lifetime) outcomes associated with the diagnosed condition.

Calculations will consider key costs and consequences, including the costs and disutility associated with having a colonoscopy. Key model input will be drawn from the RECEDE study, including

For peer review only

For peer review only - http://bmjopen.bmj.com/site/about/guidelines.xhtml

parameters related to the diagnostic characteristics of the compared options, estimates of the rate of adverse outcomes associated with colonoscopies (e.g. bowel perforation and bleeding) and CTCs, as well as primary and secondary care resource use and preference-based health-related quality of life (utility) values associated with colonoscopy. The latter information will be needed to reflect the fact that undergoing a colonoscopy, including the bowel preparation and the procedure itself is an unpleasant activity which is likely to result in a temporary decrease in quality of life. Estimates of changes in quality of life will be obtained by administering a widely used and recommended generic questionnaire (EuroQol EQ-5D-5L [21]) to a sample of 370 participants (20% of the total study sample) scheduled to undergo colonoscopy or CTC at (i) baseline (i.e. when participant consent is received) (ii) immediately prior to colonoscopy once the participant has fully completed bowel preparation, (iii) 24 hours after the colonoscopy examination, (iv) 72 hours post colonoscopy and (v) 3 weeks post colonoscopy. Use of resources, including NHS care, out of pocket payments and loss of income the participants may occur as a result of their colonoscopy will also be collected through patient questionnaires administered to the same sample of participants at 3 weeks post colonoscopy or CTC.

In line with recommendations, results will be presented in terms of total costs per additional QALY associated with FIT + VOC compared to FIT alone [22]. Deterministic and probabilistic sensitivity analyses will be undertaken to explore the robustness of the obtained results to sample variability and plausible variations in key assumptions and employed analytical methods [23, 24]. The model will also form the basis for conducting value of information analysis, which will quantify the total expected cost due to the remaining uncertainty around the decision problem [25, 26]

Peer Review and Patient & Public Involvement

The RECEDE study has been peer reviewed in the NIHR HSDR process by external reviewers and the commissioning board. This protocol has also been reviewed externally by a Trial Steering Committee and internally by the Trial Management Group (TMG). The study was also reviewed by members of

For peer review only - http://bmjopen.bmj.com/site/about/guidelines.xhtml

the GUT club (survivors of GI cancer) and the patient and public involvement in research (PPI) at the sponsor site. Both groups were supportive of the aims of RECEDE and agreed further evidence is required for the utility of FIT and VOC analysis in diagnosing SBD.

Ethics and Dissemination

Ethical approval was provided by Liverpool Central Research Ethics Committee (20/NW/0346). The study will be conducted in compliance with the principles of the ICH GCP guidelines and in accordance with all applicable regulatory guidance. The study will comply with the current Data Protection regulations and regular checks and monitoring will be undertaken by the study manger to ensure compliance.

Results from RECEDE will naturally be of profound interest to clinicians worldwide who investigate lower GI symptoms. Results can also be used by NICE to inform a revised pathway for managing participants with lower GI symptoms – to categorise high risk i.e. those with SBD versus low risk (without SBD). Those with very low risk of SBD could potentially be managed in primary care without need to refer to secondary care. RECEDE will lead to high level presentations at GI and Oncological meetings both nationally and internationally. It will also result in high quality open access manuscripts. A range of dissemination products will include annual reports, national publications, press releases through UHCW Communications Department, participant safety collaborations, presentation and talks as well as videos will ensure that all audiences can be updated. Dissemination of results to participants will be led by our PPI co-applicant and facilitated through GUTs UK which is the partner charity of the British Society of Gastroenterology. We will also engage with key stakeholders including PPI groups, local specialised colorectal Clinical Research Group, as well as Cancer Alliance groups

Author Statement

(1) Substantial contribution to design of work

For peer review only

(2) Drafting and revising the intellectual content

(3) Final approval of version to be published

(4) Agreement to be accountable for works published

Chris Bradley (1, 2, 3, 4), Siew Wan Hee (1, 2, 3, 4), Laz Andronis (1, 2, 3, 4), Krishna Persaud (1, 2, 3, 4), Mark Hull, (1, 2, 3, 4), John Todd (1, 2, 3, 4) Sian Taylor-Phillips (1, 2, 3, 4), Steve Smith (1, 2, 3, 4), Norman Waugh (1, 2, 3, 4), Rachel Constable (1, 2, 3, 4), Ramesh Arasaradnam (1, 2, 3, 4)

Figure Captions

Figure 1. Participant flow diagram for RECEDE. * indicates that the data is only being collected in 20% of participants.

Figure 2. Confidence intervals for different estimates of sensitivity for 150, 200 and 250 cases of SBD.

Figure 3. Confidence intervals for different estimates of NPV for 400,500 and 600 negative cases of SBD from 1000 samples.

For peer review only

References

[1] R. Arasaradnam, N. Bhala, C. Evans, J. Greenaway, R. Logan, I. Penman, E. Seward, B. Singh, S. Smith, J. Stephenson and N. Waugh, "Faecal immunochemical testing in the COVID-19 era: balancing risk and costs," *Lancet Gastroenterol Hepatol,* vol. 5, no. 8, pp. 717-719, 2020.

[2] NHS, "Waiting Times for Suspected and Diagnosed Cancer Patients," 2017. [Online]. Available: https://www.england.nhs.uk/statistics/wp-content/uploads/sites/2/2017/06/Cancer-Waiting-Times-Annual-Report-201617-1.pdf.

[3] M. Widlak, C. Thomas, C. Tomkins, S. Smith, N. O'Connel, S. Wurie, L. Burns, C. Harmston, C. Evans, C. Nwokolo, B. Singh and R. Arasaradnam, "Diagnostic accuracy of faecal biomarkers in detecting colorectal cancer and adenoma in symptomatic patients," *Aliment Pharmacol Ther,* vol. 45, no. 2, pp. 354-363, 2017.

[4] A. Adler, E. Solter, S. Masarwa, T. Miller-Roll, B. Abu-Libdeh, H. Khanmash, K. Najem, S. Dekadek, C. Stein-Zamir, N. Nubani, A. Kunbar, M. Assous, Y. Carmeli and M. Schwaber, "Epidemiological and Microbiological Characteristics of an Outbreak Caused by OXA-48-Producing Enterobacteriaceae in a Neonatal Intensive Care Unit in Jerusalem, Israe," *J Clin Microbiol,* vol. 51, no. 9, pp. 2926-2930, 2013.

[5] NICE, "Quantitive faecal immunochemical tests to guide referral for colorectal cancer in primary care," 2017. [Online]. Available: https://www.nice.or.uk/guidance/dg30.

[6] J. Murphy, S. Halloran and A. Gray, "Cost-effectiveness of the faecal immunochemical test at a range of positivity thresholds compared with the guaiac faecal occult blood test in the NHS Bowel Cancer Screening Programme in England," *BMJ Open,* vol. 7, no. 10, p. e017186, 2017.

[7] M. Westwood, I. Corro Ramos, S. Lang, M. Luyendijk, R. Zaim, L. Stirk, L. Maiwenn, N. Armstrong and J. Kleijnen, "Faecal immunochemical tests to triage participants with lower abdominal symptoms for suspected colorectal cancer referrals in primary care: a systematic review and cost-effectiveness analysis," *Health Technol Assess,* vol. 21, no. 33, pp. 1-234, 2017.

[8] J. Turvill, S. Mellen, L. Jeffery, S. Bevan, A. Keding and D. Turnock, "Diagnostic accuracy of one or two faecal haemoglobin and calprotectin measurements in participants with suspected colorectal cancer.," *Scand J Gastroenterol,* vol. 55, no. 2, pp. 69-76, 2018.

[9] A. Bannaga, K. F, K. Persaud and A. R, "Differentiating cancer types using a urine test for volatile organic compounds," *J Breath Res,* vol. 15, no. 1, p. 017102, 2020.

[10] H. Wright, B. AS, R. Iriarte, M. Mahmoud and A. R, "Utility of volatile organic compounds as a diagnostic tool in preterm infants," *Pediatr Res,* vol. 89, no. 2, pp. 263-268, 2021.

For peer review only - http://bmjopen.bmj.com/site/about/guidelines.xhtml

[11] S. Chandrapalan and R. Arasaradnam, "Urine as a biological modality for colorectal cancer detection," *Expert Rev Mol Diagn,* vol. 20, no. 5, pp. 489-496, 2020.

[12] J. Stonestreet, S. Chandrapalan, D. Woolley, U. Uthman and R. Arasaradnam, "Systematic review and meta-analysis : diagnostic accuracy of faecal immunochemical testing for haemoglobin (FIT) in detecting colorectal cancer for both symptomatic and screening population.," *Acta Gastroenterol Belg,* vol. 82, no. 2, pp. 291-299, 2019.

[13] M. Widlak, M. Neal, C. Thomas, C. Tomkins, B. Singh, C. Harmston, A. Wicaksono, C. Evans, S. Smith, R. Savage, J. Covington and R. Arasaradnam, "Risk stratification of symptomatic patients suspected of colorectal cancer using faecal and urinary markers," *Colorectal Disease,* vol. 20, no. 12, pp. 335-342, 2018.

[14] Tyagi, E. Daulton, A. Bannaga, R. Arasardnam and J. Covington, "Non-Invasive Detection and Staging of Colorectal Cancer Using a Portable Electronic Nose," *Sensor (Basel),* vol. 21, no. 16, p. 5440, 2021.

[15] S. Chandraplan, S. Bosch, J. Cubiella, J. Guardiola, P. Kimani, C. Mulder, K. Persaud, T. Meij, D. Altomare, H. Brenner, N. de Boer, L. Ricciardiello and R. Arasaradnam, "Systematic review with meta-analysis: volatile organic compound analysis to improve faecal immunochemical testing in the detection of colorectal cancer," *Aliment Pharmacol Ther,* vol. 54, no. 1, pp. 14-23, 2021.

[16] R. Arasaradnam, E. Westenbrink, M. McFarlane, R. Harbord, S. Chambers, N. O'Connell, C. Bailey, C. Nwokolo, D. Karna, R. Savage and J. Covington, "Differentiating coeliac disease from irritable bowel syndrome by urinary volatile organic compound analysis--a pilot study," *PLoS One,* vol. 9, no. 10, p. e107312, 2014.

[17] J. Covington, E. Westenbrink, N. Ouaret, R. Harbord, C. Bailey, N. O'Connell, J. Cullis, N. Williams, C. Nwokolo, K. Bardhan and R. Arasaradnam, "Application of a novel tool for diagnosing bile acid diarrhoea," *Sensors (Basel),* vol. 13, no. 9, pp. 11899-11912, 2013.

[18] NICE, "Suspected Cncer: recognition and referral (NICE guideline)," National Institute for Health and Care Excellence, 2015. [Online]. Available: https://www.nice.org.uk/guidance/ng12.

[19] N. Zhou X-HO and D. McClish, "Chapter 4: Estimation and hypothesis testing in a single sample," in *Statistical Methods in Diagnostic Medicine (Second Ed.)*, Hoboken, New Jersey, John Wiley & Sons, 2011, pp. 103-164.

[20] C. Bradley and A. Arasaradnam, "Making Research Happen in a Pandemic," National Institute of Health Research, 27 April 2021. [Online]. Available: https://www.nihr.ac.uk/blog/making-research-happen-in-a-pandemic/27549. [Accessed 18 October 2021].

[21] M. Herdman, C. Gudex, A. Lloyd, M. Janssen, P. Kind, D. Parkin, G. Bonsel and X. Badia, "Development and preliminary testing of the new five-level version of EQ-5D (EQ-5D-5L)," *Qual Life Res,* vol. 20, no. 10, pp. 1827-1736, 2011.

[22] NICE, "Guide to the methods of technology appraisal," 2013. [Online]. Available:

For peer review only

For peer review only - http://bmjopen.bmj.com/site/about/guidelines.xhtml

https://www.nice.org.uk/process/pmg9/chapter/foreword.

[23] T. Takai, S. Kanaoka, K. Yoshida, Y. Hamaya, M. Ikuma, N. Miura, H. Sugimura, M. Kajimura and A. Hishida, "Fecal cyclooxygenase 2 plus matrix metalloproteinase 7 mRNA assays as a marker for colorectal cancer screening," *Cancer Epidemiol Biomarkers Prev,* vol. 18, no. 6, pp. 1888-1893, 2009.

[24] M. Baietto and A. Wilson, "Electronic-nose applications for fruit identification, ripeness and quality grading. Sensors," *Sensors (Basel),* vol. 15, no. 2, pp. 899-931, 2015.

[25] L. Andronis, L. Billingham, S. Bryan, N. James and P. Barton, "A Practical Application of Value of Information and Prospective Payback of Research to Prioritize Evaluative Research. Med Decis Making.," *Med Decis Making,* vol. 36, no. 3, pp. 321-324, 2016.

[26] K. Claxton, L. Ginnelly, M. Sculpher, Z. Philips and S. Palmer, "A pilot study on the use of decision theory and value of information analysis as part of the NHS Health Technology Assessment programme.," *Health Technol Assess,* vol. 8, no. 31, pp. 1-103, 2004.

For peer review only - http://bmjopen.bmj.com/site/about/guidelines.xhtml

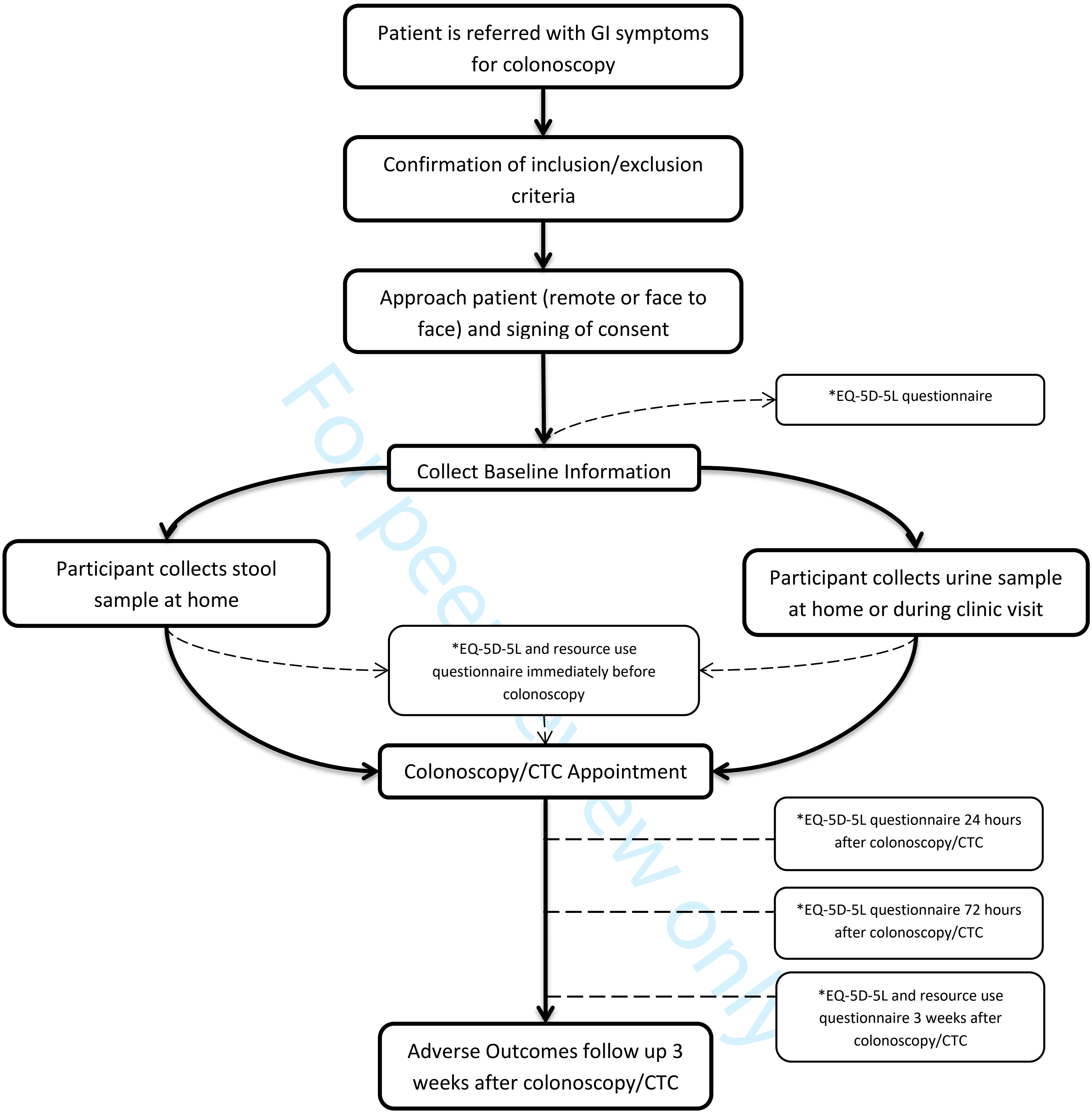

Figure 1. Participant flow diagram for RECEDE. * indicates that the data is only being collected in 20% of participants.

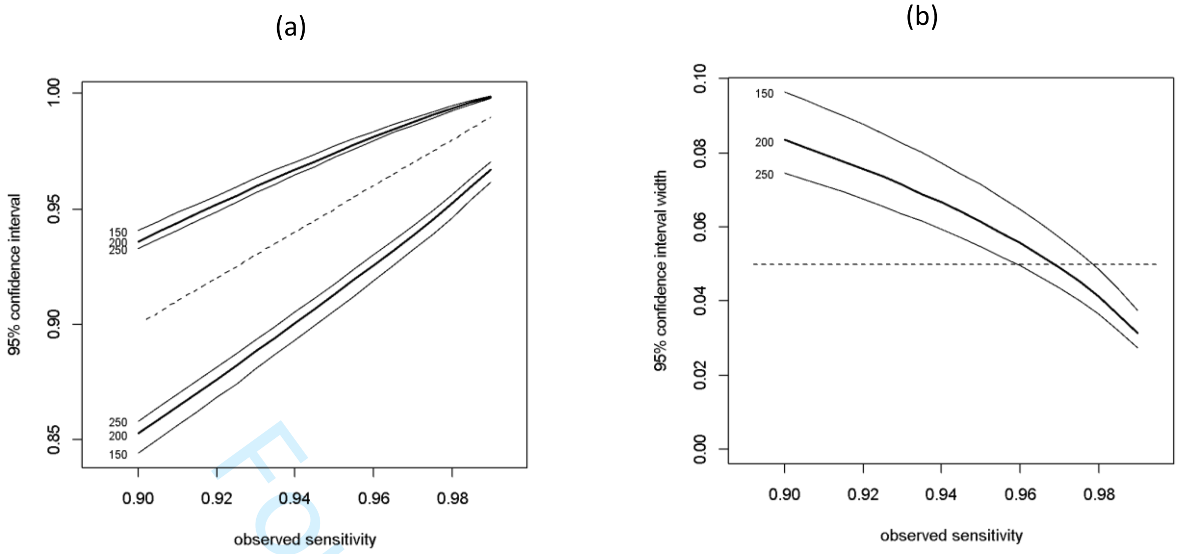

Figure 2. Confidence intervals for different estimates of sensitivity for 150, 200 and 250 cases of SBD.

For peer review only

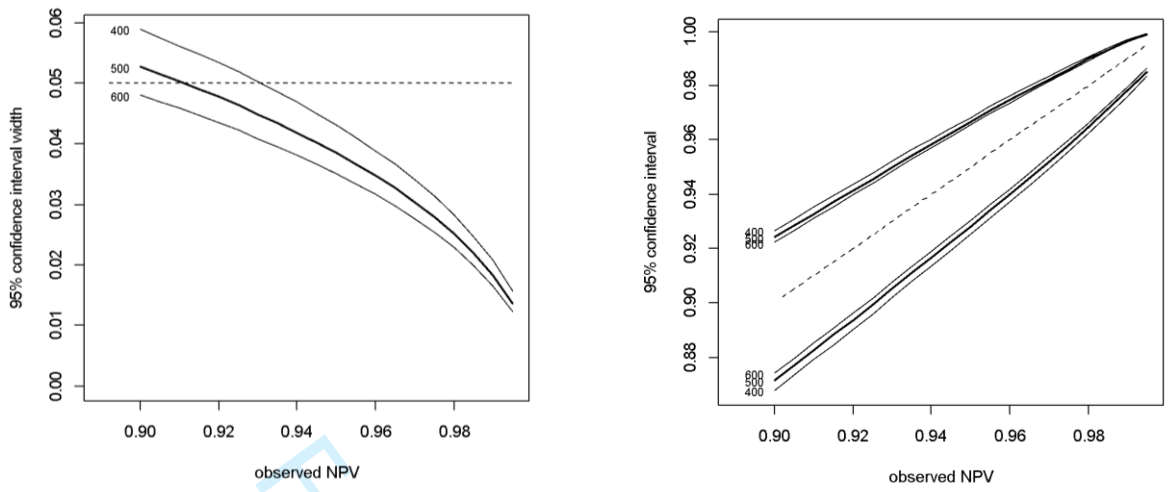

(a) (b)

Figure 3. Confidence intervals for different estimates of NPV for 400,500 and 600 negative cases of SBD from 1000 samples.

For peer review only

Reporting checklist for protocol of a clinical trial.

Based on the SPIRIT guidelines.

Instructions to authors

Complete this checklist by entering the page numbers from your manuscript where readers will find each of the items listed below.

Your article may not currently address all the items on the checklist. Please modify your text to include the missing information. If you are certain that an item does not apply, please write "n/a" and provide a short explanation.

Upload your completed checklist as an extra file when you submit to a journal.

In your methods section, say that you used the SPIRITreporting guidelines, and cite them as:

Chan A-W, Tetzlaff JM, Gøtzsche PC, Altman DG, Mann H, Berlin J, Dickersin K, Hróbjartsson A, Schulz KF, Parulekar WR, Krleža-Jerić K, Laupacis A, Moher D. SPIRIT 2013 Explanation and Elaboration: Guidance for protocols of clinical trials. BMJ. 2013;346:e7586

		Reporting Item	Page Number
**Administrative information**			
Title	#1	Descriptive title identifying the study design, population, interventions, and, if applicable, trial acronym	1
Trial registration	#2a	Trial identifier and registry name. If not yet registered, name of intended registry	2
Trial registration: data set	#2b	All items from the World Health Organization Trial Registration Data Set	n/a
Protocol version	#3	Date and version identifier	1
Funding	#4	Sources and types of financial, material, and other support	1
Roles and responsibilities: contributorship	#5a	Names, affiliations, and roles of protocol contributors	12

For peer review only

Roles and responsibilities: sponsor contact information	#5b	Name and contact information for the trial sponsor	5
Roles and responsibilities: sponsor and funder	#5c	Role of study sponsor and funders, if any, in study design; collection, management, analysis, and interpretation of data; writing of the report; and the decision to submit the report for publication, including whether they will have ultimate authority over any of these activities	n/a
Roles and responsibilities: committees	#5d	Composition, roles, and responsibilities of the coordinating centre, steering committee, endpoint adjudication committee, data management team, and other individuals or groups overseeing the trial, if applicable (see Item 21a for data monitoring committee)	11
**Introduction**			
Background and rationale	#6a	Description of research question and justification for undertaking the trial, including summary of relevant studies (published and unpublished) examining benefits and harms for each intervention	3
Background and rationale: choice of comparators	#6b	Explanation for choice of comparators	4
Objectives	#7	Specific objectives or hypotheses	5
Trial design	#8	Description of trial design including type of trial (eg, parallel group, crossover, factorial, single group), allocation ratio, and framework (eg, superiority, equivalence, non-inferiority, exploratory)	5
**Methods: Participants, interventions, and outcomes**			
Study setting	#9	Description of study settings (eg, community clinic, academic hospital) and list of countries where data will be collected. Reference to where list of study sites can be obtained	5
Eligibility criteria	#10	Inclusion and exclusion criteria for participants. If applicable, eligibility criteria for study centres and individuals who will	5

For peer review only

		perform the interventions (eg, surgeons, psychotherapists)	
Interventions: description	#11a	Interventions for each group with sufficient detail to allow replication, including how and when they will be administered	5
Interventions: modifications	#11b	Criteria for discontinuing or modifying allocated interventions for a given trial participant (eg, drug dose change in response to harms, participant request, or improving / worsening disease)	n/a
Interventions: adherance	#11c	Strategies to improve adherence to intervention protocols, and any procedures for monitoring adherence (eg, drug tablet return; laboratory tests)	n/a
Interventions: concomitant care	#11d	Relevant concomitant care and interventions that are permitted or prohibited during the trial	n/a
Outcomes	#12	Primary, secondary, and other outcomes, including the specific measurement variable (eg, systolic blood pressure), analysis metric (eg, change from baseline, final value, time to event), method of aggregation (eg, median, proportion), and time point for each outcome. Explanation of the clinical relevance of chosen efficacy and harm outcomes is strongly recommended	8
Participant timeline	#13	Time schedule of enrolment, interventions (including any run-ins and washouts), assessments, and visits for participants. A schematic diagram is highly recommended (see Figure)	figure 1
Sample size	#14	Estimated number of participants needed to achieve study objectives and how it was determined, including clinical and statistical assumptions supporting any sample size calculations	6
Recruitment	#15	Strategies for achieving adequate participant enrolment to reach target sample size	8
**Methods: Assignment of interventions (for controlled trials)**			
Allocation: sequence generation	#16a	Method of generating the allocation sequence (eg, computer-generated random numbers), and list of any factors for stratification. To reduce predictability of a random sequence, details of any planned restriction (eg, blocking) should be provided in a separate document that is unavailable to those who enrol participants or assign interventions	n/a

For peer review only

Allocation concealment mechanism	#16b	Mechanism of implementing the allocation sequence (eg, central telephone; sequentially numbered, opaque, sealed envelopes), describing any steps to conceal the sequence until interventions are assigned	n/a
Allocation: implementation	#16c	Who will generate the allocation sequence, who will enrol participants, and who will assign participants to interventions	n/a
Blinding (masking)	#17a	Who will be blinded after assignment to interventions (eg, trial participants, care providers, outcome assessors, data analysts), and how	n/a
Blinding (masking): emergency unblinding	#17b	If blinded, circumstances under which unblinding is permissible, and procedure for revealing a participant's allocated intervention during the trial	n/a
**Methods: Data collection, management, and analysis**			
Data collection plan	#18a	Plans for assessment and collection of outcome, baseline, and other trial data, including any related processes to promote data quality (eg, duplicate measurements, training of assessors) and a description of study instruments (eg, questionnaires, laboratory tests) along with their reliability and validity, if known. Reference to where data collection forms can be found, if not in the protocol	12
Data collection plan: retention	#18b	Plans to promote participant retention and complete follow-up, including list of any outcome data to be collected for participants who discontinue or deviate from intervention protocols	n/a
Data management	#19	Plans for data entry, coding, security, and storage, including any related processes to promote data quality (eg, double data entry; range checks for data values). Reference to where details of data management procedures can be found, if not in the protocol	8
Statistics: outcomes	#20a	Statistical methods for analysing primary and secondary outcomes. Reference to where other details of the statistical analysis plan can be found, if not in the protocol	8
Statistics: additional analyses	#20b	Methods for any additional analyses (eg, subgroup and adjusted analyses)	9

For peer review only

Statistics: analysis population and missing data	#20c	Definition of analysis population relating to protocol non-adherence (eg, as randomised analysis), and any statistical methods to handle missing data (eg, multiple imputation)	n/a
**Methods: Monitoring**			
Data monitoring: formal committee	#21a	Composition of data monitoring committee (DMC); summary of its role and reporting structure; statement of whether it is independent from the sponsor and competing interests; and reference to where further details about its charter can be found, if not in the protocol. Alternatively, an explanation of why a DMC is not needed	n/a
Data monitoring: interim analysis	#21b	Description of any interim analyses and stopping guidelines, including who will have access to these interim results and make the final decision to terminate the trial	n/a
Harms	#22	Plans for collecting, assessing, reporting, and managing solicited and spontaneously reported adverse events and other unintended effects of trial interventions or trial conduct	n/a
Auditing	#23	Frequency and procedures for auditing trial conduct, if any, and whether the process will be independent from investigators and the sponsor	n/a
**Ethics and dissemination**			
Research ethics approval	#24	Plans for seeking research ethics committee / institutional review board (REC / IRB) approval	2
Protocol amendments	#25	Plans for communicating important protocol modifications (eg, changes to eligibility criteria, outcomes, analyses) to relevant parties (eg, investigators, REC / IRBs, trial participants, trial registries, journals, regulators)	n/a
Consent or assent	#26a	Who will obtain informed consent or assent from potential trial participants or authorised surrogates, and how (see Item 32)	5
Consent or assent: ancillary studies	#26b	Additional consent provisions for collection and use of participant data and biological specimens in ancillary studies, if applicable	n/a
Confidentiality	#27	How personal information about potential and enrolled participants will be collected, shared, and maintained in order to protect confidentiality before, during, and after the trial	5

For peer review only

Declaration of interests	#28	Financial and other competing interests for principal investigators for the overall trial and each study site	1
Data access	#29	Statement of who will have access to the final trial dataset, and disclosure of contractual agreements that limit such access for investigators	8
Ancillary and post trial care	#30	Provisions, if any, for ancillary and post-trial care, and for compensation to those who suffer harm from trial participation	n/a
Dissemination policy: trial results	#31a	Plans for investigators and sponsor to communicate trial results to participants, healthcare professionals, the public, and other relevant groups (eg, via publication, reporting in results databases, or other data sharing arrangements), including any publication restrictions	12
Dissemination policy: authorship	#31b	Authorship eligibility guidelines and any intended use of professional writers	12
Dissemination policy: reproducible research	#31c	Plans, if any, for granting public access to the full protocol, participant-level dataset, and statistical code	12
**Appendices**			
Informed consent materials	#32	Model consent form and other related documentation given to participants and authorised surrogates	n/a
Biological specimens	#33	Plans for collection, laboratory evaluation, and storage of biological specimens for genetic or molecular analysis in the current trial and for future use in ancillary studies, if applicable	7

The SPIRIT Explanation and Elaboration paper is distributed under the terms of the Creative Commons Attribution License CC-BY-NC. This checklist was completed on 19. October 2021 using https://www.goodreports.org/, a tool made by the EQUATOR Network in collaboration with Penelope.ai

For peer review only

Section & Topic	No	Item	Reported on page #
**TITLE OR ABSTRACT**			
	**1**	Identification as a study of diagnostic accuracy using at least one measure of accuracy (such as sensitivity, specificity, predictive values, or AUC)	1
**ABSTRACT**			
	**2**	Structured summary of study design, methods, results, and conclusions (for specific guidance, see STARD for Abstracts)	3
**INTRODUCTION**			
	**3**	Scientific and clinical background, including the intended use and clinical role of the index test	4
	**4**	Study objectives and hypotheses	5
**METHODS**			
Study design	**5**	Whether data collection was planned before the index test and reference standard were performed (prospective study) or after (retrospective study)	6
Participants	**6**	Eligibility criteria	6
	**7**	On what basis potentially eligible participants were identified (such as symptoms, results from previous tests, inclusion in registry)	6
	**8**	Where and when potentially eligible participants were identified (setting, location and dates)	6
	**9**	Whether participants formed a consecutive, random or convenience series	6
Test methods	**10a**	Index test, in sufficient detail to allow replication	8
	**10b**	Reference standard, in sufficient detail to allow replication	9
	**11**	Rationale for choosing the reference standard (if alternatives exist)	7
	**12a**	Definition of and rationale for test positivity cut-offs or result categories of the index test, distinguishing pre-specified from exploratory	10
	**12b**	Definition of and rationale for test positivity cut-offs or result categories of the reference standard, distinguishing pre-specified from exploratory	n/a
	**13a**	Whether clinical information and reference standard results were available to the performers/readers of the index test	9
	**13b**	Whether clinical information and index test results were available to the assessors of the reference standard	9
Analysis	**14**	Methods for estimating or comparing measures of diagnostic accuracy	10
	**15**	How indeterminate index test or reference standard results were handled	Protocol published prospectively so n/a
	**16**	How missing data on the index test and reference standard were handled	Protocol published prospectively so n/a
	**17**	Any analyses of variability in diagnostic accuracy, distinguishing pre-specified from exploratory	10
	**18**	Intended sample size and how it was determined	7
**RESULTS**			
Participants	**19**	Flow of participants, using a diagram	Figure 1
	**20**	Baseline demographic and clinical characteristics of participants	Protocol published prospectively so n/a
	**21a**	Distribution of severity of disease in those with the target condition	Protocol published prospectively so n/a
	**21b**	Distribution of alternative diagnoses in those without the target condition	Protocol published prospectively so n/a
	**22**	Time interval and any clinical interventions between index test and reference standard	Protocol published prospectively so n/a
Test results	**23**	Cross tabulation of the index test results (or their distribution) by the results of the reference standard	Protocol published prospectively so n/a
	**24**	Estimates of diagnostic accuracy and their precision (such as 95% confidence intervals)	7
	**25**	Any adverse events from performing the index test or the reference standard	N/A
**DISCUSSION**			
	**26**	Study limitations, including sources of potential bias, statistical uncertainty, and generalisability	4
	**27**	Implications for practice, including the intended use and clinical role of the index test	5
**OTHER INFORMATION**			

For peer review only - http://bmjopen.bmj.com/site/about/guidelines.xhtml

CC BY SA

	**28**	Registration number and name of registry	3
	**29**	Where the full study protocol can be accessed	Not freely available
	**30**	Sources of funding and other support; role of funders	2

For peer review only

STARD 2015

AIM

STARD stands for “Standards for Reporting Diagnostic accuracy studies”. This list of items was developed to contribute to the completeness and transparency of reporting of diagnostic accuracy studies. Authors can use the list to write informative study reports. Editors and peer-reviewers can use it to evaluate whether the information has been included in manuscripts submitted for publication.

EXPLANATION

A **diagnostic accuracy study** evaluates the ability of one or more medical tests to correctly classify study participants as having a **target condition.** This can be a disease, a disease stage, response or benefit from therapy, or an event or condition in the future. A medical test can be an imaging procedure, a laboratory test, elements from history and physical examination, a combination of these, or any other method for collecting information about the current health status of a patient.

The test whose accuracy is evaluated is called **index test.** A study can evaluate the accuracy of one or more index tests. Evaluating the ability of a medical test to correctly classify patients is typically done by comparing the distribution of the index test results with those of the **reference standard**. The reference standard is the best available method for establishing the presence or absence of the target condition. An accuracy study can rely on one or more reference standards.

If test results are categorized as either positive or negative, the cross tabulation of the index test results against those of the reference standard can be used to estimate the **sensitivity** of the index test (the proportion of participants *with* the target condition who have a positive index test), and its **specificity** (the proportion *without* the target condition who have a negative index test). From this cross tabulation (sometimes referred to as the contingency or “2x2” table), several other accuracy statistics can be estimated, such as the positive and negative **predictive values** of the test. Confidence intervals around estimates of accuracy can then be calculated to quantify the statistical **precision** of the measurements.

If the index test results can take more than two values, categorization of test results as positive or negative requires a **test positivity cut-off**. When multiple such cut-offs can be defined, authors can report a receiver operating characteristic (ROC) curve which graphically represents the combination of sensitivity and specificity for each possible test positivity cut-off. The **area under the ROC curve** informs in a single numerical value about the overall diagnostic accuracy of the index test.

The **intended use** of a medical test can be diagnosis, screening, staging, monitoring, surveillance, prediction or prognosis. The **clinical role** of a test explains its position relative to existing tests in the clinical pathway. A replacement test, for example, replaces an existing test. A triage test is used before an existing test; an add-on test is used after an existing test.

Besides diagnostic accuracy, several other outcomes and statistics may be relevant in the evaluation of medical tests. Medical tests can also be used to classify patients for purposes other than diagnosis, such as staging or prognosis. The STARD list was not explicitly developed for these other outcomes, statistics, and study types, although most STARD items would still apply.

DEVELOPMENT

This STARD list was released in 2015. The 30 items were identified by an international expert group of methodologists, researchers, and editors. The guiding principle in the development of STARD was to select items that, when reported, would help readers to judge the potential for bias in the study, to appraise the applicability of the study findings and the validity of conclusions and recommendations. The list represents an update of the first version, which was published in 2003.

More information can be found on http://www.equator-network.org/reporting-guidelines/stard.

CC BY SA

For peer review only - http://bmjopen.bmj.com/site/about/guidelines.xhtml

BMJ Open

REducing Colonoscopies in patients without significant bowEl DiseasE – The RECEDE Study - Protocol for a Prospective Diagnostic Accuracy Study.

Journal:	*BMJ Open*
Manuscript ID	bmjopen-2021-058559.R2
Article Type:	Protocol
Date Submitted by the Author:	10-Feb-2022
Complete List of Authors:	Bradley, Christopher; University Hospitals Coventry and Warwickshire NHS Trust, Research & Development<br>Hee, Siew Wan; University of Warwick,<br>Andronis, Lazaro; University of Warwick, Warwick Medical School<br>Persaud, Krishna; The University of Manchester, Department of Chemical Engineering<br>Hull, Mark A.; Univ Bradford<br>Todd, John; University Hospitals Coventry and Warwickshire NHS Trust, Research & Development<br>Taylor-Phillips, Sian; University of Warwick, Warwick Medical School<br>Smith, Steve; University Hospitals of Coventry & Warwickshire NHS Trust, Midlands & NW Bowel Cancer Screening Hub, Hospital of St. Cross,<br>Waugh, Norman; University of Warwick, Warwick Evidence<br>Constable, Rachel; University Hospitals Coventry and Warwickshire NHS Trust, Research & Development<br>Arasaradnam, Ramesh; University Hospitals Coventry and Warwickshire NHS Trust, Gastroenterology
**Primary Subject Heading**:	Gastroenterology and hepatology
Secondary Subject Heading:	Research methods, Public health
Keywords:	Endoscopy < GASTROENTEROLOGY, Computed tomography < RADIOLOGY & IMAGING, Colorectal surgery < SURGERY

SCHOLARONE™
Manuscripts

Protocol for Publication v2.1_10.02.2022 - BMJ Open

Title: REducing Colonoscopies in patients without significant bowEl DiseasE – The RECEDE Study - Protocol for a Prospective Diagnostic Accuracy Study.

Authors:

Chris Bradley – Christopher.bradley@uhcw.nhs.uk[1]

Siew Wan Hee – S.W.Hee@warwick.ac.uk[2]

Lazaros Andronis – l.andronis@warwick.ac.uk[2]

Krishna Persaud – krishna.persaud@manchester.ac.uk[3]

Mark A Hull – m.a.hull@leeds.ac.uk[4]

John Todd[1]

Sian Taylor-Phillips – S.taylor-phillips@warwick.ac.uk[2]

Steve Smith – steve.smith@uhcw.nhs.uk[1]

Norman Waugh norman.waugh@warwick.ac.uk[2]

Rachel Constable – Rachel.constable@uhcw.nhs.uk[1]

Ramesh Arasaradnam – Ramesh.arasaradnam@uhcw.nhs.uk[1,2,5,6]

Corresponding Author

Chris Bradley

University Hospitals Coventry & Warwickshire NHS Trust, Clifford Bridge Road, Coventry, CV2 2DX

Christopher.bradley@uhcw.nhs.uk

02476966197

Author Conflicts:

[1] University Hospitals Coventry & Warwickshire NHS Trust, Coventry, England
[2] University of Warwick, Coventry, England
[3] University of Manchester, Manchester, England
[4] University of Leeds, Leeds, England
[5] Leicester Cancer Research Centre, University of Leicester, ENgland
[6] School of Health Sciences, University of Coventry, England

For peer review only - http://bmjopen.bmj.com/site/about/guidelines.xhtml

No competing interests declared

Acknowledgements:

This study is funded by the National Institute for Health Research (NIHR) [Health Service and Delivery Research (project reference 127800)]. The views expressed are those of the authors and not necessarily those of the NIHR or the Department of Health and Social Care.

In memory of John Todd, deceased 17th April 2021

Key Words:

Colonoscopy, Computed Tomography Colonography, Volatile Organic Compounds, Faecal Immunochemical Testing, Colorectal Cancer

Word Count:

3566

For peer review only

Abstract

Introduction: Demand for colonoscopies and computed tomography colonography (CTC) is exceeding capacity in NHS Trusts. In many patients colonoscopies and CTCs show no significant bowel disease (SBD). Faecal Immunochemical Testing (FIT) is being introduced to prioritise patients for colonoscopies but is insufficient to identify non-SBD patients meaning colonoscopy and CTC demand remains high. The RECEDE study aims to test urine volatile organic compound (VOC) analysis alongside FIT to improve detection of SBD and to reduce the number of colonoscopies and CTCs.

Methods and Analysis: This is a multi-centre, prospective diagnostic accuracy study evaluating whether stool FIT plus urine VOC compared to stool FIT alone improves detection of SBD in patients referred for colonoscopy or CTC due to persistent lower gastrointestinal (GI) symptoms. To ensure SBD is not missed, the dual test requires a high sensitivity, set at 97% with 95% confidence interval width of 5%. Our assumption is that to achieve this sensitivity requires 200 participants with SBD. Further assuming 19% of all participants will have SBD and 55% of all participants will return both stool and urine samples we will recruit 1915 participants. The thresholds for FIT and VOC results diagnosing SBD have been pre-set. If either FIT or VOC exceeds the respective threshold, the participant will be classed as having suspected SBD. As an exploratory analysis we will be testing different thresholds. The reference comparator will be a complete colonoscopy or CTC. Secondary outcomes will look at optimising the FIT and VOC thresholds for SBD detection. An economic evaluation, using a denovo decision analytic model, will be carried out determine the costs, benefits and overall cost-effectiveness of FIT + VOC versus FIT followed by colonoscopy.

Ethics and Dissemination: Ethical approval was obtained by Liverpool Central Research Ethics Committee (20/NW/0346). RECEDE is registered on Clinicaltrials.gov (NCT04516785) & ISRCTN (14982373)

For peer review only - http://bmjopen.bmj.com/site/about/guidelines.xhtml

Article Summary

Strengths and Limitations of the Study

- Simple observational trial design that does not affect the standard care of participants
- The study can be conducted entirely remotely.
- Reliance on patients collecting their samples at home and posting/bringing their samples back to the hospital may result in a higher proportion of missed samples as there is a greater chance of patients forgetting.
- Recruitment rate is dependent on COVID 19 as referral rates drop when COVID 19 cases are high [1]

Introduction

There is currently a disparity between demand and available resources for colonoscopies and CTCs within the NHS. Before the COVID-19 pandemic, around 300,000 patients were being referred annually to NHS trusts suspected of having colorectal cancer (CRC) and this number was rising [2]. Most patients referred with suspected CRC are offered an invasive colonoscopy or CTC examination, but only 30% of these participants have significant bowel disease (SBD) [3]. SBD is defined as CRC, adenomatous polyps or inflammatory bowel disease [4]. The remaining 70% have a normal examination with 30% of those having functional conditions such as irritable bowel syndrome (IBS).

Set against this there will remain a capacity shortfall for colonoscopies and CTCs for the foreseeable future. This limits capacity of the NHS to extend CRC detection within the Bowel Cancer Screening Programme (BCSP) or to investigate those who present through the emergency department. The imbalance between capacity and demand, coupled with a lack of an accurate triage test makes it difficult for the NHS to stratify patients who present with bowel symptoms to those most at risk of

For peer review only - http://bmjopen.bmj.com/site/about/guidelines.xhtml

SBD. In the COVID-19 era, faecal immunochemical testing (FIT) is being introduced to triage patients who present with lower GI symptoms [1]. However almost all patients still receive a colonoscopy or alternative colonic imaging at some time and the optimum threshold for FIT has yet to be determined. The National Institute for Health and Care Excellence (NICE) have recommended 10μ gHb/g faeces [5], whereas the BCSP have set a cut off of 120μgHb/g faeces [6]. Even at the lower threshold some SBD cases can be missed [3, 7, 8]. Previous data have shown that FIT alone (at a threshold of 7μgHb/g faeces) has a sensitivity of 80%, 53%and 86% for CRC, adenomatous polyps and inflammatory bowel disease (IBD) respectively [3]. A lower FIT threshold may reduce the number of false negatives but it will increase the number of unnecessary colonoscopies and CTCs in patients with no abnormalities (false positives). Moreover it will always miss cases of SBD where there is no bleeding, and a high proportion of malignant tumours never bleed.

Thus, this study will test urine volatile organic compound (VOC) analysis alongside FIT to improve detection of SBD. VOCs are produced by metabolic responses to inflammation in the presence of illness, therefore they are disease specific [9, 10, 11]. Previous work has identified key metabolites in inflammatory GI disease [12] and, on its own, VOC analysis has a sensitivity of 80%, 92% and 86% for CRC, adenomatous polyps and IBD respectively [13, 14]. When FIT and VOC are used in combination (FIT followed by VOC in the FIT negative), sensitivity of CRC improves from 80% to 97%, which is similar to the results of colonoscopy and CTC [15, 13]. Existing studies have already demonstrated the utility of VOC analysis in detecting IBD, coeliac disease and bile acid diarrhoea whilst also being negative in functional conditions such as IBS [16, 17]. Many patients with slightly raised FIT are found to have no SBD and we will examine the value of VOC in this group to see if a negative VOC can rule out SBD.

Therefore, the objective of this study is to investigate whether the combination of FIT and VOC analysis compared to FIT alone improves detection of SBD in patients who present with lower GI symptoms. . This could be by both improving sensitivity in patients with FIT < 10 and but also

For peer review only - http://bmjopen.bmj.com/site/about/guidelines.xhtml

possibly by improving specificity in patients with slightly raised FIT but negative VOC. If correct, fewer patients will be referred for colonoscopies and CTCs, freeing up capacity and reducing NHS costs, whilst reducing disutility in patients who do not benefit from an invasive examination. Within the COVID-19 era, this has added importance. If the proposed method of dual testing proves effective then not only will hospital capacity be improved but also fewer patients will be required to undergo colonoscopies or CTCs; streamlining the patient pathway and reducing the risk of viral transmission if colonoscopy generates aerosols.

Methods and Analysis

Study Design and Procedures

This is a multi-centre (UK only), prospective diagnostic accuracy study, sponsored by the University Hospitals Coventry & Warwickshire (UHCW) to compare dual testing of stool FIT plus urine VOC markers compared to stool FIT alone in the detection of SBD. Participants will be recruited when referred to secondary care for investigation of lower bowel symptoms, after informed consent is received. **Inclusion criteria** will be: patients referred non-urgently or urgently (fulfilling the national criteria for referral – NICE NG12) [18] with lower GI symptoms and determined by the overseeing clinician to require colonoscopy or CTC; minimum age of 18; able to provide informed consent, and have the ability to return both stool and urine samples. **Exclusion criteria** are those who are pregnant. Participants will be recruited over the phone or face-to-face during a clinic visit which precedes their colonoscopy or CTC. Participants will be asked to provide a stool sample and urine sample at least 24 hours before commencing their bowel cleansing medication. Stool samples will be collected at home by the participants using FIT collection kits and posted directly to the analysis site. If the participant has already completed a FIT during their referral pathway, we will use this data if the time between the FIT and their colonoscopy is less than 4 weeks, otherwise we will request that the participant to provide another FIT. Urine samples will be returned to hospitals and frozen at -80°C pending future analysis. Urine samples can either be collected during a clinic visit that precedes

For peer review only - http://bmjopen.bmj.com/site/about/guidelines.xhtml

their colonoscopy or CTC, or collected at the home of the participant and brought back by the participant. We will aim for the time between the urine sample being produced and the urine sample being frozen at -80ºC to be less than 4 hours, otherwise we will ask participants to freeze their urine samples in their home freezer. For the purposes of the economic analysis, a subset of participants (20% of total) undergoing colonoscopy or CTC will also be asked to complete questionnaires at five time points surrounding their examination. This will help determine the disutility and costs associated with undergoing colonoscopy or CTC, which will be used as inputs in the economic model (see section Economic Evaluation below). Figure 1 show's a participant flow diagram once enrolled in the RECEDE study.

Sample Size Calculation

To replace colonoscopy – the current gold standard for diagnosing bowel conditions – and/or CTC, FIT and VOC analysis requires a very high sensitivity to avoid false negatives. High specificity is also desirable, to reduce false positives. Based on our previous study, the sensitivity of FIT and VOC analysis to detect CRC was 97%, comparable to colonoscopy. Therefore, the sample size was determined to achieve a sensitivity of at least 97% with a 95% confidence interval width of 5% (Zhou-Li method [19] as described in Box 1). The required number of participants with SBD to achieve this sensitivity is 200. Figure 2 shows confidence intervals for different observed sensitivity cases of SBD (150, 200, 250). Figure 3 shows the widths of the confidence interval limits are narrower and wider when the observed sensitivity is above or below 97% [13], respectively. This sample size is also robust to negative predictive value (NPV), an outcome that is of interest as a high NPV suggests that participants without SBD would avoid having colonoscopy examination. Assuming the prevalence of SBD in the study population is 19%, then 1053 participants are required. Based on our previous study [13], the return rate of both stool and urine samples that are eligible for analysis is about 55% resulting in a total required sample size of 1915 participants across multiple UK sites within a 24 month period.

For peer review only

For peer review only - http://bmjopen.bmj.com/site/about/guidelines.xhtml

The Zhou Li confidence interval is $\left(\frac{e^{LL}}{1+e^{LL}},\frac{e^{UL}}{1+e^{UL}}\right)\left(\frac{e^{LL}}{1+e^{LL}},\frac{e^{UL}}{1+e^{UL}}\right)$ where $LL=\ln\left(\frac{\hat{Se}}{1-\hat{Se}}\right)-\frac{1}{\sqrt{n\hat{Se}(1-\hat{Se})}}g^{-1}(z_{1-\alpha/2})$, $UL=\ln\left(\frac{\hat{Se}}{1-\hat{Se}}\right)-\frac{1}{\sqrt{n\hat{Se}(1-\hat{Se})}}g^{-1}(z_{\alpha/2})$, $g^{-1}(x)=-\sqrt{n}\left(\frac{6}{\hat{\gamma}}\right)\left\{\left[1-\frac{\hat{\gamma}}{2}\left(\frac{x}{\sqrt{n}}-\frac{\hat{\gamma}}{6n}\right)\right]^{1/3}-1\right\}$ and $\hat{\gamma}=\frac{1-2\hat{Se}}{\hat{Se}(1-\hat{Se})}$.

Box 1: Sample size determination based on confidence interval method

Stool Sample Analysis

Stool samples will be analysed for traces of haemoglobin in the faeces using a HM-JACKarc. Participants samples will be defined as having suspected SBD if the value is >10μgHb/g faeces. Any previous FIT results collected to triage patients can be sourced from patient records if the test is <4 weeks before their colonoscopy or CTC.

Urine VOC Analysis

Participants will be asked to collect duplicate urine samples in universal sterilin pots. Urine samples will be transported to the analysis site using dry ice to maintain the cold chain. All urine samples will be analysed using the 'electronic nose' [16] technique. Briefly, this technique enables separation of VOCs between disease groups based on chemical fingerprint pattern rather than specific chemical analysis. This allows screening of populations rapidly using chemo metric data analysis (information extracted from chemical systems) and receiver operator curves (ROC) to distinguish one population from another, with ROCs being used to measure the appropriate thresholds to be set to distinguish samples as having SBD. A sample which has a ROC with an area under the curve of 0.63 or greater will be defined as having suspected SBD.

A subset of 100 urine samples will also be analysed using a gas-chromatography mass spectroscopy system. This technique separates complex mixtures of chemicals based on their interaction with a retentive layer, resulting in chemicals eluding out of the gas-chromatography at different times.

For peer review only

For peer review only - http://bmjopen.bmj.com/site/about/guidelines.xhtml

These individual chemicals are then ionised and the mass of the resultant fragments measured. The analysis will be split into two stages. Firstly, a subset of SBD positive and SBD negative samples will be screened to identify up to 8 VOCs that are prominent in SBD positive samples. Once identified, a headspace gas chromatography/mass spectrometry (HSGC-MS) method will be developed to detect, separate and quantify the target VOCs selected in stage one within the remaining samples. This method will highlight whether specific VOCs present more frequently in SBD positive samples.

Adoption of Study due to COVID pandemic – remote delivery

In order to continue recruiting through the COVID-19 pandemic, an innovative study design was adopted whereby participant involvement shifted to an entirely remote approach. This allowed the study to continue recruiting through the peak waves of COVID-19 when many other studies were forced to temporarily pause [20].

Reference Test

The reference test is the final report from the colonoscopy or CTC examination and confirmatory histology report. Biopsies are required as per national guidance [5] in the investigation of participants with lower GI symptoms even in the absence of macroscopic abnormalities. Those who perform the reference test will have no knowledge of the results of the index tests, and vice versa.

Data Management

Data from the study will be stored on an online validated GCP compliant electronic data capture (EDC) system. Individual user access will be provided only to members of the research team who require it for their role. No personal data will be uploaded to the EDC. Participant identification codes will be used to ensure pseudo-anonymization. Following the resolution of queries, the database will be locked and exported to the trial statistician for analysis. Access to the final trial dataset will only be made available to those who require it for the analysis.

For peer review only - http://bmjopen.bmj.com/site/about/guidelines.xhtml

Primary Outcome Analysis

The primary outcome is diagnostic accuracy (sensitivity, specificity, negative predictive value (NPV) and positive predictive value (PPV)) of the dual index test FIT and VOC analysis. The 'or' rule will be utilised for the primary analysis whereby a FIT result of >10 µgHb/g faeces OR a VOC result with predicted probability >0.63 implies that the participant is suspected of having SBD and needs to be referred for colonoscopy or CTC. We will report 2x2 tables of test accuracy of the dual test by the reference standard (final report from the colonoscopy examination and confirmatory histology report of SBD which is either colon cancer, colorectal adenomas or inflammatory bowel disease), alongside summary measures of sensitivity, specificity, NPV and PPV, and their 95% confidence intervals. These test accuracy estimates will be used in the economic model.

We will analyse the combined FIT+VOC test results as both parallel (results from both tests are interpreted in combination) and serial testing (VOC analysis only performed if FIT is negative). The 'or' rule will be considered in the interpretation of both parallel and serial tests where the diagnosis is positive when either test is positive, and negative when both results are negative. In the serial test, if the FIT is positive, then the diagnosis is positive. If FIT is negative we will use the result from VOC analysis and if that is positive then the diagnosis is positive. Mathematically, both methods should give the same diagnostic results but will make a difference in the economic model.

For peer review only

Secondary Outcome Analyses

Secondary outcomes include: ROC of FIT plus VOC to develop an optimum thresholds for SBD detection; sensitivity, specificity, PPV and NPV estimates and their corresponding 95% CI for each individual condition of SBD (colorectal cancer, colorectal adenomas, and inflammatory bowel disease) by each individual test and combination rules; compliance rates (return of samples); the types of SBD and microscopic colitis, and the number of cases missed by CTC, FIT and VOC analyses; potential number of avoidable colonoscopies and

For peer review only - http://bmjopen.bmj.com/site/about/guidelines.xhtml

CTCs in patients without SBD; total NHS and personal social services (PSS) costs, and total quality of adjusted life years (QALY) associated with each option. To find the optimal thresholds of FIT and VOC we will first plot the ROC curves of both tests individually to find the individual optimal thresholds (the one that gives the greatest specificity whilst achieving 97% sensitivity). Secondly, we will plot combinations of the two tests in the ROC space and similarly, choose the combination of thresholds which has greatest specificity whilst achieving the required 97% sensitivity to avoid colonoscopy. The combined thresholds of FIT and VOC in the ROC space are calculated by combining every unit of FIT (from 10 to 120µgHb/g faeces) with every unit of the predicted probability of VOC (from 0.63 to 1). Both the 'or' and 'and' rules will be explored when combining the FIT and VOC results to define a case as either SBD positive or SBD negative. If both tests are in discordant then the diagnosis is negative. For each of the thresholds that we derive from the ROC curves, we will also report the NPV estimates. This will produce an overestimate of test accuracy, which we will use as a sensitivity analysis in the economic model, as a more optimistic assumption to demonstrate the range of uncertainty.

Economic Evaluation

An economic analysis will be carried out to determine the costs, benefits and overall cost-effectiveness of FIT + VOC versus FIT alone in selecting patients for colonoscopy or CTC amongst those with GI symptoms referred for investigation. The analysis will be carried out primarily from the perspective of NHS and it will employ a *de novo* analytic model built as part of this study. In line with recent literature [7], it is envisaged that the model will employ a multi-part structure, consisting of a decision tree to evaluate short-term cost and consequences accruing at the diagnosis stage followed by a state-transition model to capture the long-term (lifetime) outcomes associated with the diagnosed condition.

Calculations will consider key costs and consequences, including the costs and disutility associated with having a colonoscopy. Key model input will be drawn from the RECEDE study, including

For peer review only - http://bmjopen.bmj.com/site/about/guidelines.xhtml

parameters related to the diagnostic characteristics of the compared options, estimates of the rate of adverse outcomes associated with colonoscopies (e.g. bowel perforation and bleeding) and CTCs, as well as primary and secondary care resource use and preference-based health-related quality of life (utility) values associated with colonoscopy. The latter information will be needed to reflect the fact that undergoing a colonoscopy, including the bowel preparation and the procedure itself is an unpleasant activity which is likely to result in a temporary decrease in quality of life. Estimates of changes in quality of life will be obtained by administering a widely used and recommended generic questionnaire (EuroQol EQ-5D-5L [21]) to a sample of 370 participants (20% of the total study sample) scheduled to undergo colonoscopy or CTC at (i) baseline (i.e. when participant consent is received) (ii) immediately prior to colonoscopy once the participant has fully completed bowel preparation, (iii) 24 hours after the colonoscopy examination, (iv) 72 hours post colonoscopy and (v) 3 weeks post colonoscopy. Use of resources, including NHS care, out of pocket payments and loss of income the participants may occur as a result of their colonoscopy will also be collected through patient questionnaires administered to the same sample of participants at 3 weeks post colonoscopy or CTC.

In line with recommendations, results will be presented in terms of total costs per additional QALY associated with FIT + VOC compared to FIT alone [22]. Deterministic and probabilistic sensitivity analyses will be undertaken to explore the robustness of the obtained results to sample variability and plausible variations in key assumptions and employed analytical methods [23, 24]. The model will also form the basis for conducting value of information analysis, which will quantify the total expected cost due to the remaining uncertainty around the decision problem [25, 26]

Peer Review and Patient & Public Involvement

The RECEDE study has been peer reviewed in the NIHR HSDR process by external reviewers and the commissioning board. This protocol has also been reviewed externally by a Trial Steering Committee and internally by the Trial Management Group (TMG). The study was also reviewed by members of

For peer review only - http://bmjopen.bmj.com/site/about/guidelines.xhtml

the GUT club (survivors of GI cancer) and the patient and public involvement in research (PPI) at the sponsor site. Both groups were supportive of the aims of RECEDE and agreed further evidence is required for the utility of FIT and VOC analysis in diagnosing SBD.

Ethics and Dissemination

Ethical approval was provided by Liverpool Central Research Ethics Committee (20/NW/0346). The study will be conducted in compliance with the principles of the ICH GCP guidelines and in accordance with all applicable regulatory guidance. The study will comply with the current Data Protection regulations and regular checks and monitoring will be undertaken by the study manger to ensure compliance.

Results from RECEDE will naturally be of profound interest to clinicians worldwide who investigate lower GI symptoms. Results can also be used by NICE to inform a revised pathway for managing participants with lower GI symptoms – to categorise high risk i.e. those with SBD versus low risk (without SBD). Those with very low risk of SBD could potentially be managed in primary care without need to refer to secondary care. RECEDE will lead to high level presentations at GI and Oncological meetings both nationally and internationally. It will also result in high quality open access manuscripts. A range of dissemination products will include annual reports, national publications, press releases through UHCW Communications Department, participant safety collaborations, presentation and talks as well as videos will ensure that all audiences can be updated. Dissemination of results to participants will be led by our PPI co-applicant and facilitated through GUTs UK which is the partner charity of the British Society of Gastroenterology. We will also engage with key stakeholders including PPI groups, local specialised colorectal Clinical Research Group, as well as Cancer Alliance groups

Author Statement

(1) Substantial contribution to design of work

For peer review only

(2) Drafting and revising the intellectual content

(3) Final approval of version to be published

(4) Agreement to be accountable for works published

Chris Bradley (1, 2, 3, 4), Siew Wan Hee (1, 2, 3, 4), Laz Andronis (1, 2, 3, 4), Krishna Persaud (1, 2, 3, 4), Mark Hull, (1, 2, 3, 4), John Todd (1, 2, 3, 4) Sian Taylor-Phillips (1, 2, 3, 4), Steve Smith (1, 2, 3, 4), Norman Waugh (1, 2, 3, 4), Rachel Constable (1, 2, 3, 4), Ramesh Arasaradnam (1, 2, 3, 4)

Figure Captions

Figure 1. Participant flow diagram for RECEDE. * indicates that the data is only being collected in 20% of participants.

Figure 2. Confidence intervals for different estimates of sensitivity for 150, 200 and 250 cases of SBD.

Figure 3. Confidence intervals for different estimates of NPV for 400,500 and 600 negative cases of SBD from 1000 samples.

For peer review only

273

274 **References**

275

[1] R. Arasaradnam, N. Bhala, C. Evans, J. Greenaway, R. Logan, I. Penman, E. Seward, B. Singh, S. Smith, J. Stephenson and N. Waugh, "Faecal immunochemical testing in the COVID-19 era: balancing risk and costs," *Lancet Gastroenterol Hepatol,* vol. 5, no. 8, pp. 717-719, 2020.

[2] NHS, "Waiting Times for Suspected and Diagnosed Cancer Patients," 2017. [Online]. Available: https://www.england.nhs.uk/statistics/wp-content/uploads/sites/2/2017/06/Cancer-Waiting-Times-Annual-Report-201617-1.pdf.

[3] M. Widlak, C. Thomas, C. Tomkins, S. Smith, N. O'Connel, S. Wurie, L. Burns, C. Harmston, C. Evans, C. Nwokolo, B. Singh and R. Arasaradnam, "Diagnostic accuracy of faecal biomarkers in detecting colorectal cancer and adenoma in symptomatic patients," *Aliment Pharmacol Ther,* vol. 45, no. 2, pp. 354-363, 2017.

[4] A. Adler, E. Solter, S. Masarwa, T. Miller-Roll, B. Abu-Libdeh, H. Khanmash, K. Najem, S. Dekadek, C. Stein-Zamir, N. Nubani, A. Kunbar, M. Assous, Y. Carmeli and M. Schwaber, "Epidemiological and Microbiological Characteristics of an Outbreak Caused by OXA-48-Producing Enterobacteriaceae in a Neonatal Intensive Care Unit in Jerusalem, Israe," *J Clin Microbiol,* vol. 51, no. 9, pp. 2926-2930, 2013.

[5] NICE, "Quantitive faecal immunochemical tests to guide referral for colorectal cancer in primary care," 2017. [Online]. Available: https://www.nice.or.uk/guidance/dg30.

[6] J. Murphy, S. Halloran and A. Gray, "Cost-effectiveness of the faecal immunochemical test at a range of positivity thresholds compared with the guaiac faecal occult blood test in the NHS Bowel Cancer Screening Programme in England," *BMJ Open,* vol. 7, no. 10, p. e017186, 2017.

[7] M. Westwood, I. Corro Ramos, S. Lang, M. Luyendijk, R. Zaim, L. Stirk, L. Maiwenn, N. Armstrong and J. Kleijnen, "Faecal immunochemical tests to triage participants with lower abdominal symptoms for suspected colorectal cancer referrals in primary care: a systematic review and cost-effectiveness analysis," *Health Technol Assess,* vol. 21, no. 33, pp. 1-234, 2017.

[8] J. Turvill, S. Mellen, L. Jeffery, S. Bevan, A. Keding and D. Turnock, "Diagnostic accuracy of one or two faecal haemoglobin and calprotectin measurements in participants with suspected colorectal cancer.," *Scand J Gastroenterol,* vol. 55, no. 2, pp. 69-76, 2018.

[9] A. Bannaga, K. F, K. Persaud and A. R, "Differentiating cancer types using a urine test for volatile organic compounds," *J Breath Res,* vol. 15, no. 1, p. 017102, 2020.

[10] H. Wright, B. AS, R. Iriarte, M. Mahmoud and A. R, "Utility of volatile organic compounds as a diagnostic tool in preterm infants," *Pediatr Res,* vol. 89, no. 2, pp. 263-268, 2021.

For peer review only - http://bmjopen.bmj.com/site/about/guidelines.xhtml

[11] S. Chandrapalan and R. Arasaradnam, "Urine as a biological modality for colorectal cancer detection," *Expert Rev Mol Diagn,* vol. 20, no. 5, pp. 489-496, 2020.

[12] J. Stonestreet, S. Chandrapalan, D. Woolley, U. Uthman and R. Arasaradnam, "Systematic review and meta-analysis : diagnostic accuracy of faecal immunochemical testing for haemoglobin (FIT) in detecting colorectal cancer for both symptomatic and screening population.," *Acta Gastroenterol Belg,* vol. 82, no. 2, pp. 291-299, 2019.

[13] M. Widlak, M. Neal, C. Thomas, C. Tomkins, B. Singh, C. Harmston, A. Wicaksono, C. Evans, S. Smith, R. Savage, J. Covington and R. Arasaradnam, "Risk stratification of symptomatic patients suspected of colorectal cancer using faecal and urinary markers," *Colorectal Disease,* vol. 20, no. 12, pp. 335-342, 2018.

[14] Tyagi, E. Daulton, A. Bannaga, R. Arasardnam and J. Covington, "Non-Invasive Detection and Staging of Colorectal Cancer Using a Portable Electronic Nose," *Sensor (Basel),* vol. 21, no. 16, p. 5440, 2021.

[15] S. Chandraplan, S. Bosch, J. Cubiella, J. Guardiola, P. Kimani, C. Mulder, K. Persaud, T. Meij, D. Altomare, H. Brenner, N. de Boer, L. Ricciardiello and R. Arasaradnam, "Systematic review with meta-analysis: volatile organic compound analysis to improve faecal immunochemical testing in the detection of colorectal cancer," *Aliment Pharmacol Ther,* vol. 54, no. 1, pp. 14-23, 2021.

[16] R. Arasaradnam, E. Westenbrink, M. McFarlane, R. Harbord, S. Chambers, N. O'Connell, C. Bailey, C. Nwokolo, D. Karna, R. Savage and J. Covington, "Differentiating coeliac disease from irritable bowel syndrome by urinary volatile organic compound analysis--a pilot study," *PLoS One,* vol. 9, no. 10, p. e107312, 2014.

[17] J. Covington, E. Westenbrink, N. Ouaret, R. Harbord, C. Bailey, N. O'Connell, J. Cullis, N. Williams, C. Nwokolo, K. Bardhan and R. Arasaradnam, "Application of a novel tool for diagnosing bile acid diarrhoea," *Sensors (Basel),* vol. 13, no. 9, pp. 11899-11912, 2013.

[18] NICE, "Suspected Cancer: recognition and referral (NICE guideline)," National Institute for Health and Care Excellence, 2015. [Online]. Available: https://www.nice.org.uk/guidance/ng12.

[19] N. Zhou X-HO and D. McClish, "Chapter 4: Estimation and hypothesis testing in a single sample," in *Statistical Methods in Diagnostic Medicine (Second Ed.)*, Hoboken, New Jersey, John Wiley & Sons, 2011, pp. 103-164.

[20] C. Bradley and A. Arasaradnam, "Making Research Happen in a Pandemic," National Institute of Health Research, 27 April 2021. [Online]. Available: https://www.nihr.ac.uk/blog/making-research-happen-in-a-pandemic/27549. [Accessed 18 October 2021].

[21] M. Herdman, C. Gudex, A. Lloyd, M. Janssen, P. Kind, D. Parkin, G. Bonsel and X. Badia, "Development and preliminary testing of the new five-level version of EQ-5D (EQ-5D-5L)," *Qual Life Res,* vol. 20, no. 10, pp. 1827-1736, 2011.

[22] NICE, "Guide to the methods of technology appraisal," 2013. [Online]. Available:

For peer review only - http://bmjopen.bmj.com/site/about/guidelines.xhtml

https://www.nice.org.uk/process/pmg9/chapter/foreword.

[23] T. Takai, S. Kanaoka, K. Yoshida, Y. Hamaya, M. Ikuma, N. Miura, H. Sugimura, M. Kajimura and A. Hishida, "Fecal cyclooxygenase 2 plus matrix metalloproteinase 7 mRNA assays as a marker for colorectal cancer screening," *Cancer Epidemiol Biomarkers Prev,* vol. 18, no. 6, pp. 1888-1893, 2009.

[24] M. Baietto and A. Wilson, "Electronic-nose applications for fruit identification, ripeness and quality grading. Sensors," *Sensors (Basel),* vol. 15, no. 2, pp. 899-931, 2015.

[25] L. Andronis, L. Billingham, S. Bryan, N. James and P. Barton, "A Practical Application of Value of Information and Prospective Payback of Research to Prioritize Evaluative Research. Med Decis Making.," *Med Decis Making,* vol. 36, no. 3, pp. 321-324, 2016.

[26] K. Claxton, L. Ginnelly, M. Sculpher, Z. Philips and S. Palmer, "A pilot study on the use of decision theory and value of information analysis as part of the NHS Health Technology Assessment programme.," *Health Technol Assess,* vol. 8, no. 31, pp. 1-103, 2004.

276

277

For peer review only

For peer review only - http://bmjopen.bmj.com/site/about/guidelines.xhtml

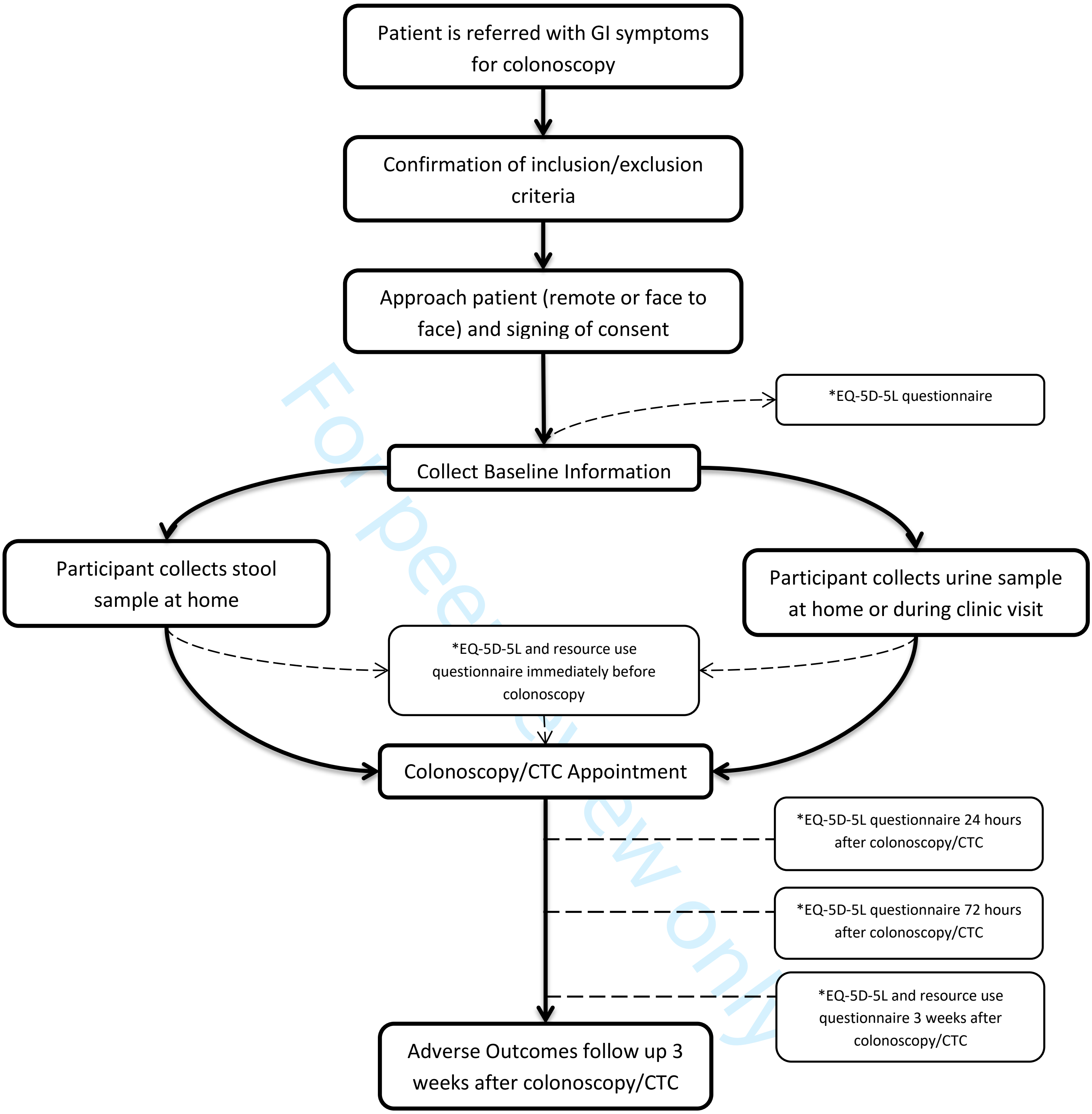

Figure 1. Participant flow diagram for RECEDE. * indicates that the data is only being collected in 20% of participants.

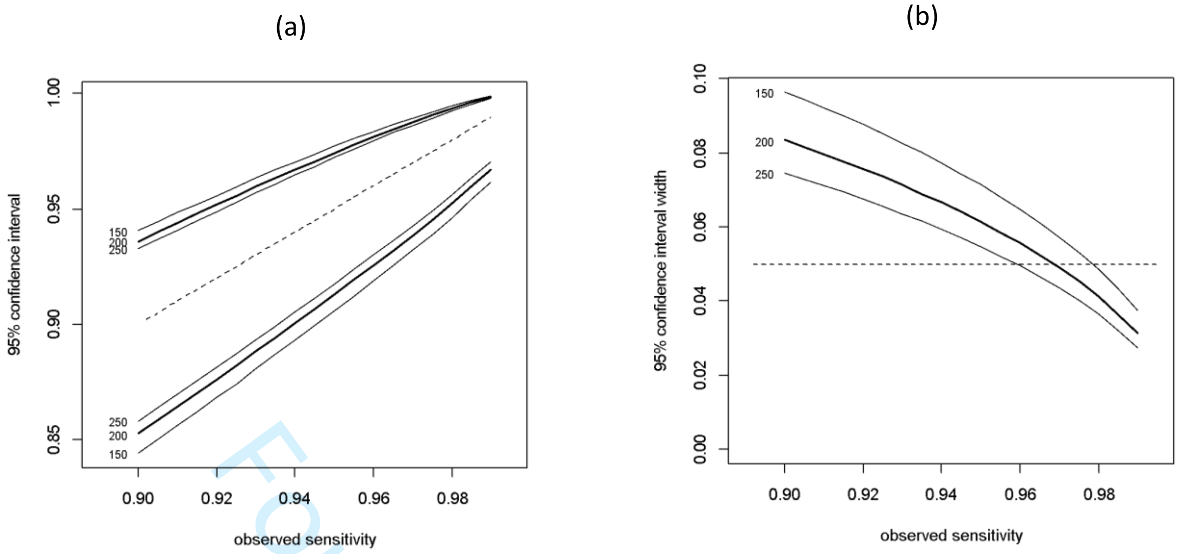

Figure 2. Confidence intervals for different estimates of sensitivity for 150, 200 and 250 cases of SBD.

For peer review only

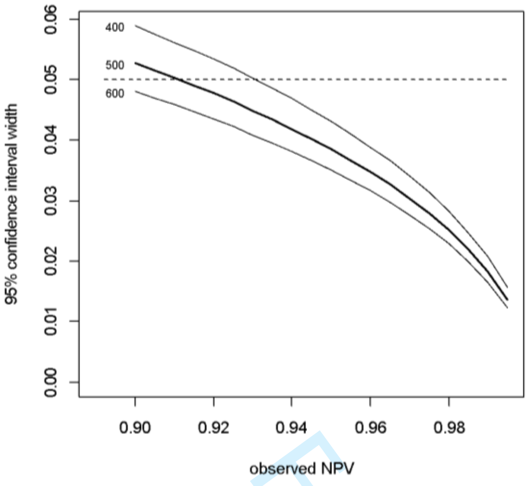

(a)

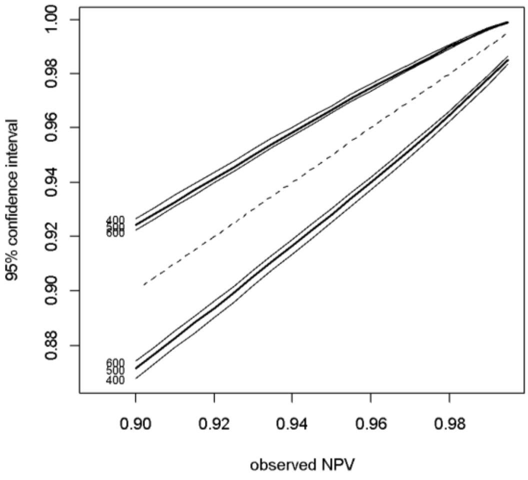

(b)

Figure 3. Confidence intervals for different estimates of NPV for 400,500 and 600 negative cases of SBD from 1000 samples.

For peer review only

Reporting checklist for protocol of a clinical trial.

Based on the SPIRIT guidelines.

Instructions to authors

Complete this checklist by entering the page numbers from your manuscript where readers will find each of the items listed below.

Your article may not currently address all the items on the checklist. Please modify your text to include the missing information. If you are certain that an item does not apply, please write "n/a" and provide a short explanation.

Upload your completed checklist as an extra file when you submit to a journal.

In your methods section, say that you used the SPIRITreporting guidelines, and cite them as:

Chan A-W, Tetzlaff JM, Gøtzsche PC, Altman DG, Mann H, Berlin J, Dickersin K, Hróbjartsson A, Schulz KF, Parulekar WR, Krleža-Jerić K, Laupacis A, Moher D. SPIRIT 2013 Explanation and Elaboration: Guidance for protocols of clinical trials. BMJ. 2013;346:e7586

		Reporting Item	Page Number
**Administrative information**			
Title	#1	Descriptive title identifying the study design, population, interventions, and, if applicable, trial acronym	1
Trial registration	#2a	Trial identifier and registry name. If not yet registered, name of intended registry	2
Trial registration: data set	#2b	All items from the World Health Organization Trial Registration Data Set	n/a
Protocol version	#3	Date and version identifier	1
Funding	#4	Sources and types of financial, material, and other support	1
Roles and responsibilities: contributorship	#5a	Names, affiliations, and roles of protocol contributors	12

For peer review only

Roles and responsibilities: sponsor contact information	#5b	Name and contact information for the trial sponsor	5
Roles and responsibilities: sponsor and funder	#5c	Role of study sponsor and funders, if any, in study design; collection, management, analysis, and interpretation of data; writing of the report; and the decision to submit the report for publication, including whether they will have ultimate authority over any of these activities	n/a
Roles and responsibilities: committees	#5d	Composition, roles, and responsibilities of the coordinating centre, steering committee, endpoint adjudication committee, data management team, and other individuals or groups overseeing the trial, if applicable (see Item 21a for data monitoring committee)	11
**Introduction**			
Background and rationale	#6a	Description of research question and justification for undertaking the trial, including summary of relevant studies (published and unpublished) examining benefits and harms for each intervention	3
Background and rationale: choice of comparators	#6b	Explanation for choice of comparators	4
Objectives	#7	Specific objectives or hypotheses	5
Trial design	#8	Description of trial design including type of trial (eg, parallel group, crossover, factorial, single group), allocation ratio, and framework (eg, superiority, equivalence, non-inferiority, exploratory)	5
**Methods: Participants, interventions, and outcomes**			
Study setting	#9	Description of study settings (eg, community clinic, academic hospital) and list of countries where data will be collected. Reference to where list of study sites can be obtained	5
Eligibility criteria	#10	Inclusion and exclusion criteria for participants. If applicable, eligibility criteria for study centres and individuals who will	5

For peer review only

		perform the interventions (eg, surgeons, psychotherapists)	
Interventions: description	#11a	Interventions for each group with sufficient detail to allow replication, including how and when they will be administered	5
Interventions: modifications	#11b	Criteria for discontinuing or modifying allocated interventions for a given trial participant (eg, drug dose change in response to harms, participant request, or improving / worsening disease)	n/a
Interventions: adherance	#11c	Strategies to improve adherence to intervention protocols, and any procedures for monitoring adherence (eg, drug tablet return; laboratory tests)	n/a
Interventions: concomitant care	#11d	Relevant concomitant care and interventions that are permitted or prohibited during the trial	n/a
Outcomes	#12	Primary, secondary, and other outcomes, including the specific measurement variable (eg, systolic blood pressure), analysis metric (eg, change from baseline, final value, time to event), method of aggregation (eg, median, proportion), and time point for each outcome. Explanation of the clinical relevance of chosen efficacy and harm outcomes is strongly recommended	8
Participant timeline	#13	Time schedule of enrolment, interventions (including any run-ins and washouts), assessments, and visits for participants. A schematic diagram is highly recommended (see Figure)	figure 1
Sample size	#14	Estimated number of participants needed to achieve study objectives and how it was determined, including clinical and statistical assumptions supporting any sample size calculations	6
Recruitment	#15	Strategies for achieving adequate participant enrolment to reach target sample size	8
**Methods: Assignment of interventions (for controlled trials)**			
Allocation: sequence generation	#16a	Method of generating the allocation sequence (eg, computer-generated random numbers), and list of any factors for stratification. To reduce predictability of a random sequence, details of any planned restriction (eg, blocking) should be provided in a separate document that is unavailable to those who enrol participants or assign interventions	n/a

For peer review only

Allocation concealment mechanism	#16b	Mechanism of implementing the allocation sequence (eg, central telephone; sequentially numbered, opaque, sealed envelopes), describing any steps to conceal the sequence until interventions are assigned	n/a
Allocation: implementation	#16c	Who will generate the allocation sequence, who will enrol participants, and who will assign participants to interventions	n/a
Blinding (masking)	#17a	Who will be blinded after assignment to interventions (eg, trial participants, care providers, outcome assessors, data analysts), and how	n/a
Blinding (masking): emergency unblinding	#17b	If blinded, circumstances under which unblinding is permissible, and procedure for revealing a participant's allocated intervention during the trial	n/a
**Methods: Data collection, management, and analysis**			
Data collection plan	#18a	Plans for assessment and collection of outcome, baseline, and other trial data, including any related processes to promote data quality (eg, duplicate measurements, training of assessors) and a description of study instruments (eg, questionnaires, laboratory tests) along with their reliability and validity, if known. Reference to where data collection forms can be found, if not in the protocol	12
Data collection plan: retention	#18b	Plans to promote participant retention and complete follow-up, including list of any outcome data to be collected for participants who discontinue or deviate from intervention protocols	n/a
Data management	#19	Plans for data entry, coding, security, and storage, including any related processes to promote data quality (eg, double data entry; range checks for data values). Reference to where details of data management procedures can be found, if not in the protocol	8
Statistics: outcomes	#20a	Statistical methods for analysing primary and secondary outcomes. Reference to where other details of the statistical analysis plan can be found, if not in the protocol	8
Statistics: additional analyses	#20b	Methods for any additional analyses (eg, subgroup and adjusted analyses)	9

For peer review only

Statistics: analysis population and missing data	#20c	Definition of analysis population relating to protocol non-adherence (eg, as randomised analysis), and any statistical methods to handle missing data (eg, multiple imputation)	n/a
**Methods: Monitoring**			
Data monitoring: formal committee	#21a	Composition of data monitoring committee (DMC); summary of its role and reporting structure; statement of whether it is independent from the sponsor and competing interests; and reference to where further details about its charter can be found, if not in the protocol. Alternatively, an explanation of why a DMC is not needed	n/a
Data monitoring: interim analysis	#21b	Description of any interim analyses and stopping guidelines, including who will have access to these interim results and make the final decision to terminate the trial	n/a
Harms	#22	Plans for collecting, assessing, reporting, and managing solicited and spontaneously reported adverse events and other unintended effects of trial interventions or trial conduct	n/a
Auditing	#23	Frequency and procedures for auditing trial conduct, if any, and whether the process will be independent from investigators and the sponsor	n/a
**Ethics and dissemination**			
Research ethics approval	#24	Plans for seeking research ethics committee / institutional review board (REC / IRB) approval	2
Protocol amendments	#25	Plans for communicating important protocol modifications (eg, changes to eligibility criteria, outcomes, analyses) to relevant parties (eg, investigators, REC / IRBs, trial participants, trial registries, journals, regulators)	n/a
Consent or assent	#26a	Who will obtain informed consent or assent from potential trial participants or authorised surrogates, and how (see Item 32)	5
Consent or assent: ancillary studies	#26b	Additional consent provisions for collection and use of participant data and biological specimens in ancillary studies, if applicable	n/a
Confidentiality	#27	How personal information about potential and enrolled participants will be collected, shared, and maintained in order to protect confidentiality before, during, and after the trial	5

For peer review only

Declaration of interests	#28	Financial and other competing interests for principal investigators for the overall trial and each study site	1
Data access	#29	Statement of who will have access to the final trial dataset, and disclosure of contractual agreements that limit such access for investigators	8
Ancillary and post trial care	#30	Provisions, if any, for ancillary and post-trial care, and for compensation to those who suffer harm from trial participation	n/a
Dissemination policy: trial results	#31a	Plans for investigators and sponsor to communicate trial results to participants, healthcare professionals, the public, and other relevant groups (eg, via publication, reporting in results databases, or other data sharing arrangements), including any publication restrictions	12
Dissemination policy: authorship	#31b	Authorship eligibility guidelines and any intended use of professional writers	12
Dissemination policy: reproducible research	#31c	Plans, if any, for granting public access to the full protocol, participant-level dataset, and statistical code	12
**Appendices**			
Informed consent materials	#32	Model consent form and other related documentation given to participants and authorised surrogates	n/a
Biological specimens	#33	Plans for collection, laboratory evaluation, and storage of biological specimens for genetic or molecular analysis in the current trial and for future use in ancillary studies, if applicable	7

The SPIRIT Explanation and Elaboration paper is distributed under the terms of the Creative Commons Attribution License CC-BY-NC. This checklist was completed on 19. October 2021 using https://www.goodreports.org/, a tool made by the EQUATOR Network in collaboration with Penelope.ai

For peer review only

Section & Topic	No	Item	Reported on page #
**TITLE OR ABSTRACT**			
	1	Identification as a study of diagnostic accuracy using at least one measure of accuracy (such as sensitivity, specificity, predictive values, or AUC)	1
**ABSTRACT**			
	2	Structured summary of study design, methods, results, and conclusions (for specific guidance, see STARD for Abstracts)	3
**INTRODUCTION**			
	3	Scientific and clinical background, including the intended use and clinical role of the index test	4
	4	Study objectives and hypotheses	5
**METHODS**			
Study design	5	Whether data collection was planned before the index test and reference standard were performed (prospective study) or after (retrospective study)	6
Participants	6	Eligibility criteria	6
	7	On what basis potentially eligible participants were identified (such as symptoms, results from previous tests, inclusion in registry)	6
	8	Where and when potentially eligible participants were identified (setting, location and dates)	6
	9	Whether participants formed a consecutive, random or convenience series	6
Test methods	10a	Index test, in sufficient detail to allow replication	8
	10b	Reference standard, in sufficient detail to allow replication	9
	11	Rationale for choosing the reference standard (if alternatives exist)	7
	12a	Definition of and rationale for test positivity cut-offs or result categories of the index test, distinguishing pre-specified from exploratory	10
	12b	Definition of and rationale for test positivity cut-offs or result categories of the reference standard, distinguishing pre-specified from exploratory	n/a
	13a	Whether clinical information and reference standard results were available to the performers/readers of the index test	9
	13b	Whether clinical information and index test results were available to the assessors of the reference standard	9
Analysis	14	Methods for estimating or comparing measures of diagnostic accuracy	10
	15	How indeterminate index test or reference standard results were handled	Protocol published prospectively so n/a
	16	How missing data on the index test and reference standard were handled	Protocol published prospectively so n/a
	17	Any analyses of variability in diagnostic accuracy, distinguishing pre-specified from exploratory	10
	18	Intended sample size and how it was determined	7
**RESULTS**			
Participants	19	Flow of participants, using a diagram	Figure 1
	20	Baseline demographic and clinical characteristics of participants	Protocol published prospectively so n/a
	21a	Distribution of severity of disease in those with the target condition	Protocol published prospectively so n/a
	21b	Distribution of alternative diagnoses in those without the target condition	Protocol published prospectively so n/a
	22	Time interval and any clinical interventions between index test and reference standard	Protocol published prospectively so n/a
Test results	23	Cross tabulation of the index test results (or their distribution) by the results of the reference standard	Protocol published prospectively so n/a
	24	Estimates of diagnostic accuracy and their precision (such as 95% confidence intervals)	7
	25	Any adverse events from performing the index test or the reference standard	N/A
**DISCUSSION**			
	26	Study limitations, including sources of potential bias, statistical uncertainty, and generalisability	4
	27	Implications for practice, including the intended use and clinical role of the index test	5
**OTHER INFORMATION**			

For peer review only - http://bmjopen.bmj.com/site/about/guidelines.xhtml

CC BY SA

	**28**	Registration number and name of registry	3
	**29**	Where the full study protocol can be accessed	Not freely available
	**30**	Sources of funding and other support; role of funders	2

For peer review only

STARD 2015

AIM

STARD stands for "Standards for Reporting Diagnostic accuracy studies". This list of items was developed to contribute to the completeness and transparency of reporting of diagnostic accuracy studies. Authors can use the list to write informative study reports. Editors and peer-reviewers can use it to evaluate whether the information has been included in manuscripts submitted for publication.

EXPLANATION

A **diagnostic accuracy study** evaluates the ability of one or more medical tests to correctly classify study participants as having a **target condition.** This can be a disease, a disease stage, response or benefit from therapy, or an event or condition in the future. A medical test can be an imaging procedure, a laboratory test, elements from history and physical examination, a combination of these, or any other method for collecting information about the current health status of a patient.

The test whose accuracy is evaluated is called **index test.** A study can evaluate the accuracy of one or more index tests. Evaluating the ability of a medical test to correctly classify patients is typically done by comparing the distribution of the index test results with those of the **reference standard**. The reference standard is the best available method for establishing the presence or absence of the target condition. An accuracy study can rely on one or more reference standards.

If test results are categorized as either positive or negative, the cross tabulation of the index test results against those of the reference standard can be used to estimate the **sensitivity** of the index test (the proportion of participants *with* the target condition who have a positive index test), and its **specificity** (the proportion *without* the target condition who have a negative index test). From this cross tabulation (sometimes referred to as the contingency or "2x2" table), several other accuracy statistics can be estimated, such as the positive and negative **predictive values** of the test. Confidence intervals around estimates of accuracy can then be calculated to quantify the statistical **precision** of the measurements.

If the index test results can take more than two values, categorization of test results as positive or negative requires a **test positivity cut-off**. When multiple such cut-offs can be defined, authors can report a receiver operating characteristic (ROC) curve which graphically represents the combination of sensitivity and specificity for each possible test positivity cut-off. The **area under the ROC curve** informs in a single numerical value about the overall diagnostic accuracy of the index test.

The **intended use** of a medical test can be diagnosis, screening, staging, monitoring, surveillance, prediction or prognosis. The **clinical role** of a test explains its position relative to existing tests in the clinical pathway. A replacement test, for example, replaces an existing test. A triage test is used before an existing test; an add-on test is used after an existing test.

Besides diagnostic accuracy, several other outcomes and statistics may be relevant in the evaluation of medical tests. Medical tests can also be used to classify patients for purposes other than diagnosis, such as staging or prognosis. The STARD list was not explicitly developed for these other outcomes, statistics, and study types, although most STARD items would still apply.

DEVELOPMENT

This STARD list was released in 2015. The 30 items were identified by an international expert group of methodologists, researchers, and editors. The guiding principle in the development of STARD was to select items that, when reported, would help readers to judge the potential for bias in the study, to appraise the applicability of the study findings and the validity of conclusions and recommendations. The list represents an update of the first version, which was published in 2003.

More information can be found on http://www.equator-network.org/reporting-guidelines/stard.

CC BY SA

For peer review only
